# Supplementary material for: The Native Bees of Texas: Evaluating the Benefits of a Public Engagement Course
Source: Insects. 2021 Aug 5;12(8):702. doi: 10.3390/insects12080702 (PMC8396608; doi:10.3390/insects12080702)
Supplement: Supplementary file 1 [file insects-12-00702-s001.zip › Supplementary-Figures_Native-Bees-Texas.pdf]

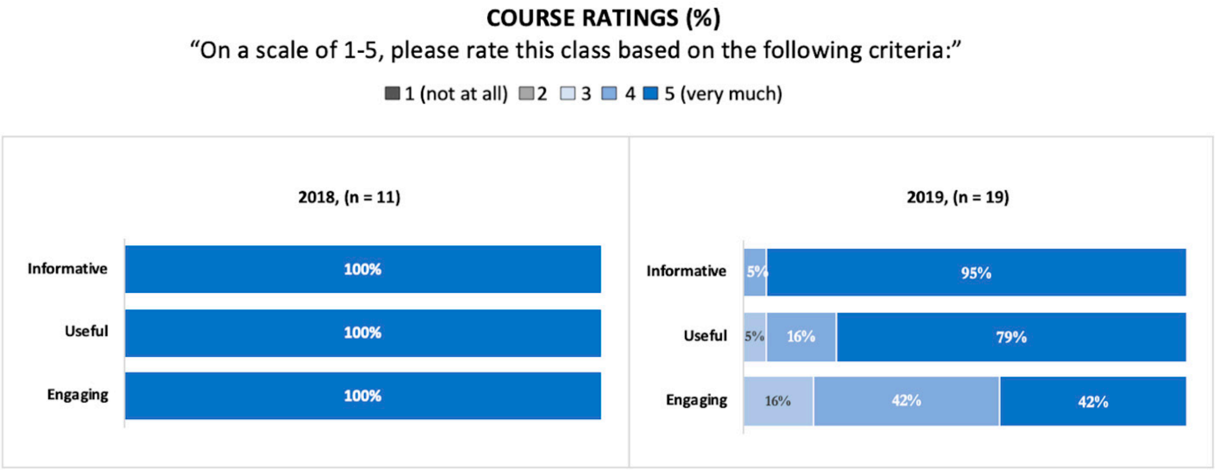

Figure S1. Overall Course Ratings
Participants rated the course based on three criteria, how informative, useful, and engaging it was for them. Mean ratings, on a 5-point Semantic Differential Scale (1, Not at all to 5, Very much), expressed as percent of each criterion. "On a scale of 1 to 5, the class was..." (see Supplementary Table S8).
