# Supplementary material for: The Native Bees of Texas: Evaluating the Benefits of a Public Engagement Course
Source: Insects. 2021 Aug 5;12(8):702. doi: 10.3390/insects12080702 (PMC8396608; doi:10.3390/insects12080702)
Supplement: Supplementary file 1 [file insects-12-00702-s001.zip › Supplementary-Schemata_Native-Bees-Texas-Compress.pdf]

Scheme S1.

Classroom instruction and educational material demos

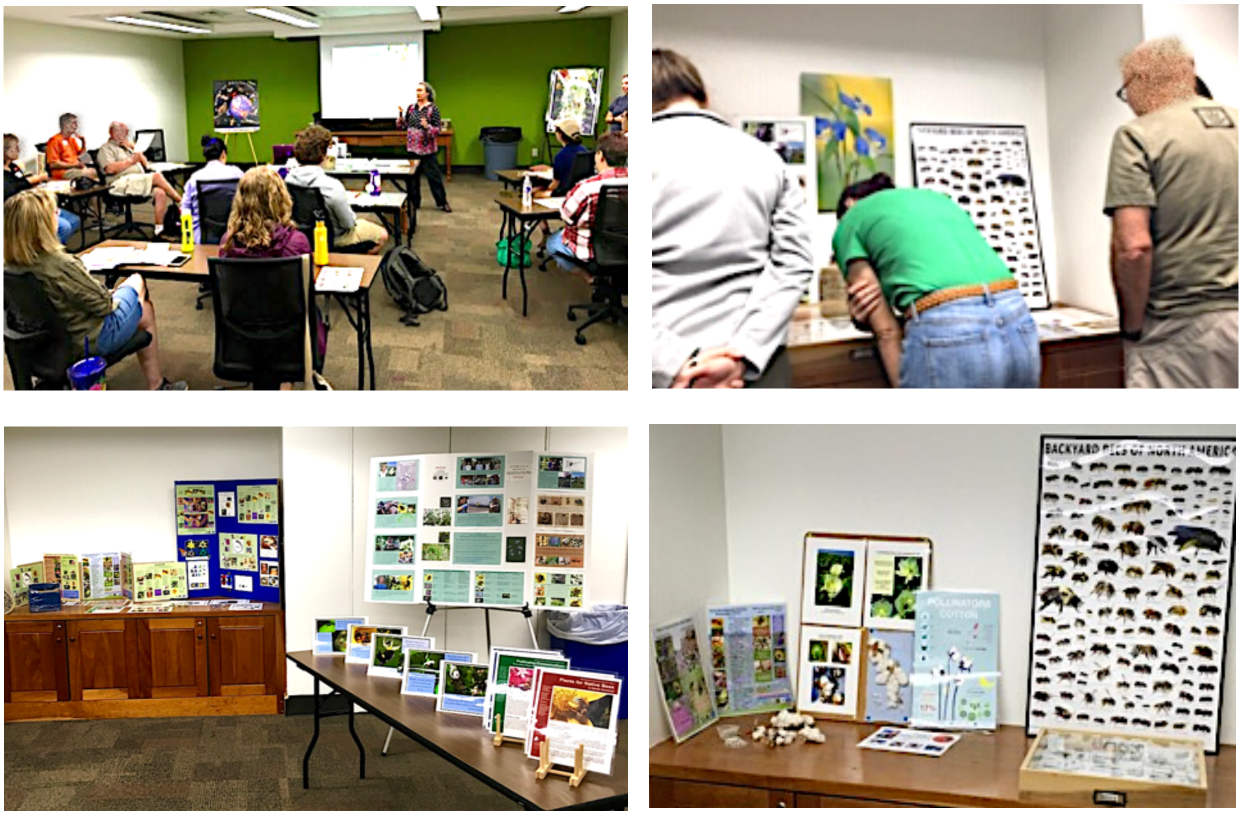

Lab layout: Stereo microscopes and loupes with pinned insects and labeled photos

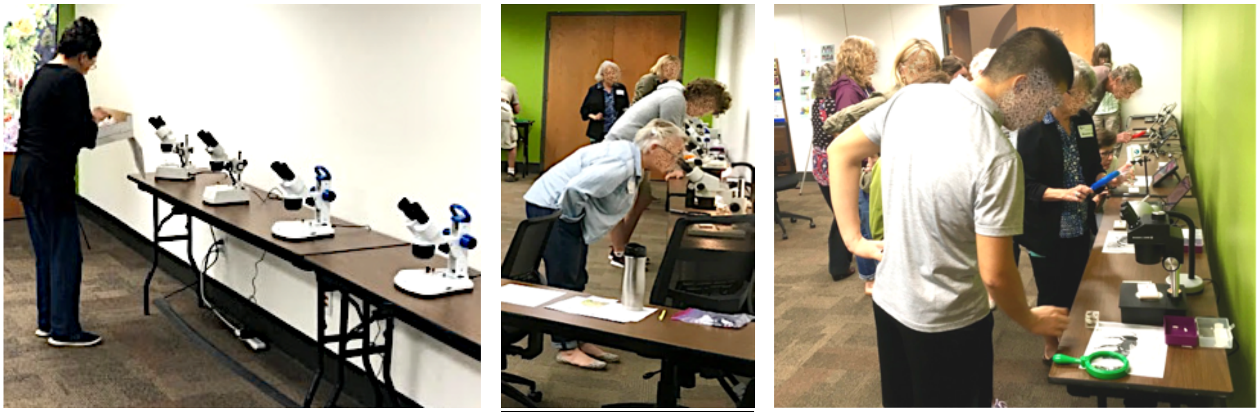

Photos Carlos Torres Verdín

Scheme S2 A-I. Educational material supplemented each lecture and lab topic (in parenthesis)

A.	Bee family cladogram	(Evolutionary classification of 6 North American bee families)
B.	Bee family/common name table	(Taxonomy of native bees, from common to uncommon in Texas)
C.	Bee ID Guide for Central Texas	(ID/diversity, lab & field observations)
D.	Bees of Central Texas Gardens	(ID/diversity, field observation. Most common bees in C. TX gardens)
E.	Bees of the Wildflower Center	(ID/diversity, field observation of bees on-site native landscapes)
F.	Tips to Improve Native Bee Habitat	(Best management practices to conserve native bee habitat)
G.	Pollinator Habitat Conservation	(Guidelines to manage and conserve native pollinator habitat)
H.	TX Prairie Plants for Native Bees	(Top ten native plant families, genera, & species / bee food resources)
I.	Native Landscapes for Native Bees	(Tips for establishing native plant landscapes for bee food & nesting)

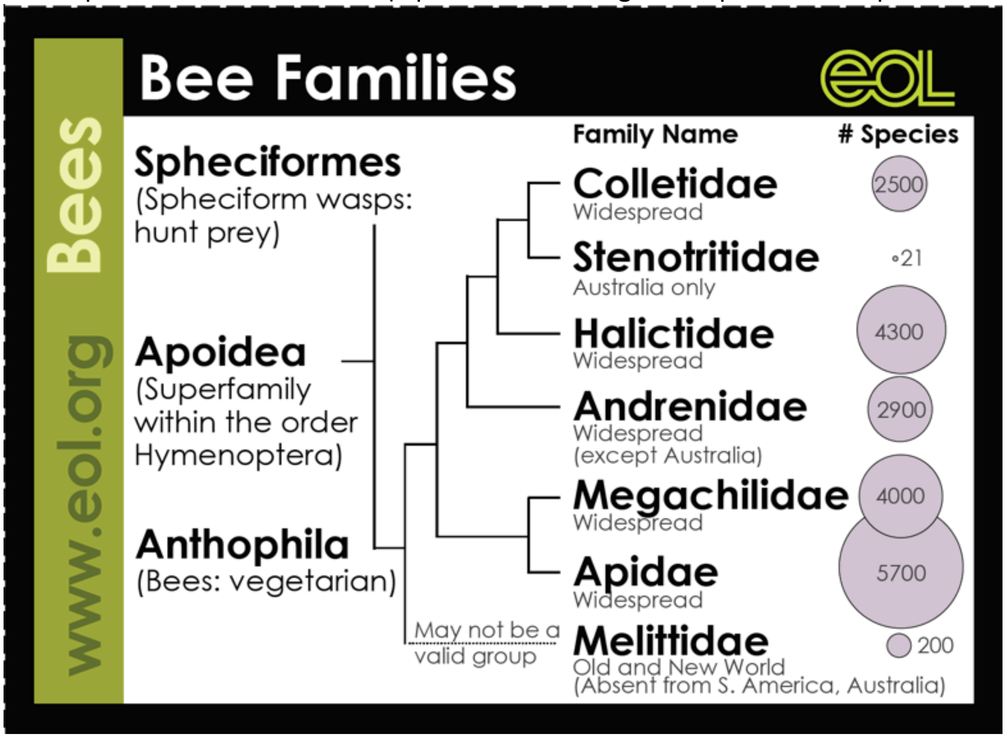

S2A

Rykken & Holmes Encyclopedia of Life https://education.eol.org/observer_cards/bee_cards_ebook.pdf

Bee Families in Texas and North America

|----------------- Long-tongued ------------------| |--------------------------- Short-tongued -----------------------------|

APIDAE	*MEGACHILIDAE*	*HALICTIDAE*	*ANDRENIDAE*	*COLLETIDAE*	*MELITTIDAE*
Bumble	Leafcutters	Sweat:	Miners:	Plasterers	Oil-collectors
Carpenters	Carders	- tiny dark	- small	Cellophane/ Polyester	Cuckoos
Longhorn	Masons	- striped	- large	Cuckoos	
Diggers/Miners	Cuckoos	- green			
Sunflower Cactus		Alkali			
Squash		Cuckoos			
Honey bee					
Cuckoos					

Note: Long or short tongued are relative terms (see references 1, 2, 3 below)

S2B

S2C. Bees of Central Texas Guide (ID/diversity, lab/field observations) (4-page document)

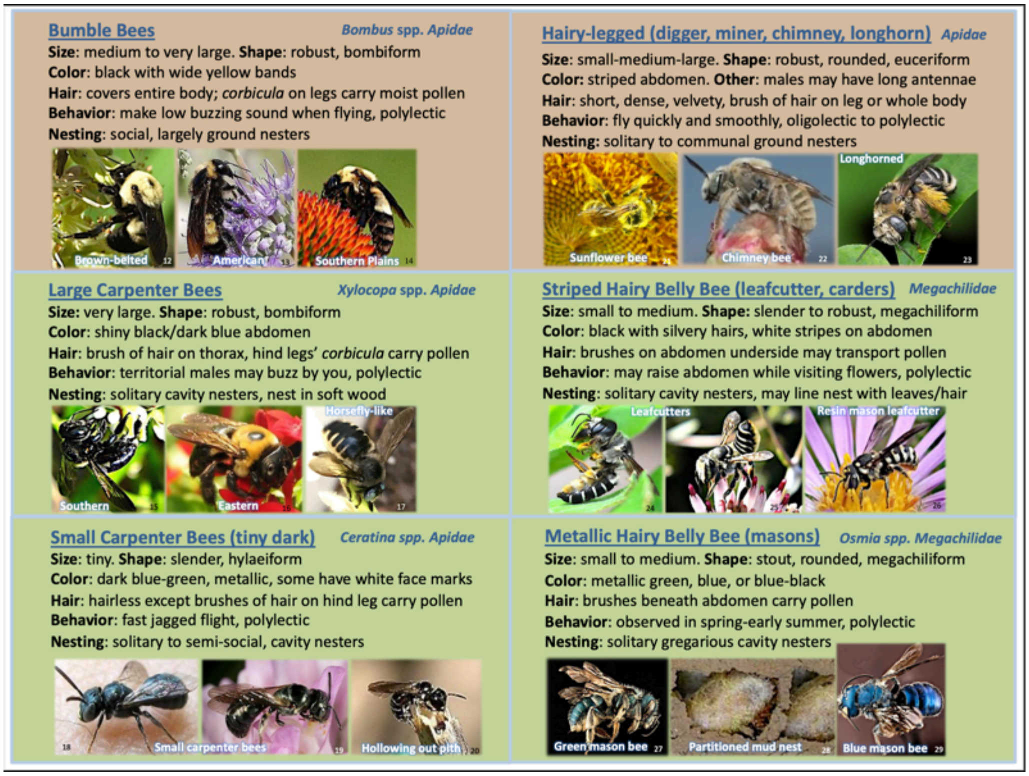

http://w3.biosci.utexas.edu/jha/wp-content/uploads//Texas-Bee-ID-Guide.pdf

S2D. Bees of Central Texas Gardens (ID/diversity, most common native bees in Central TX gardens)

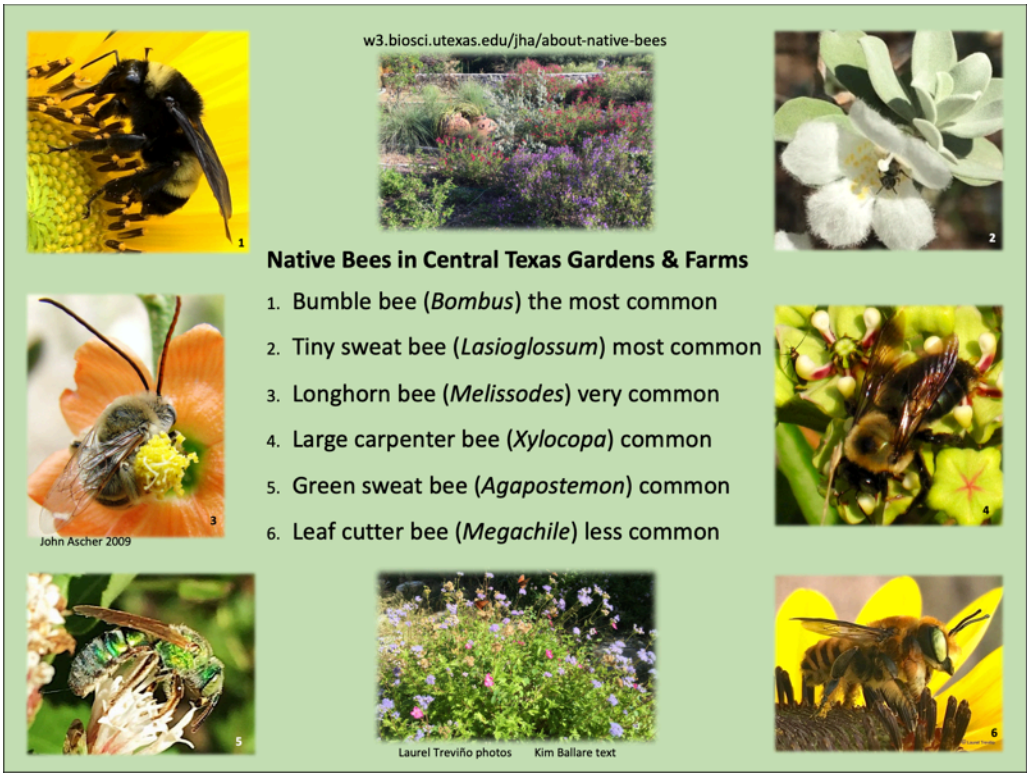

http://w3.biosci.utexas.edu/jha/wp-content/uploads//TX-Garden-Native-Bees.pdf

S2E. Local bee fauna - ID guide for bees of UT-LBJWC botanical garden

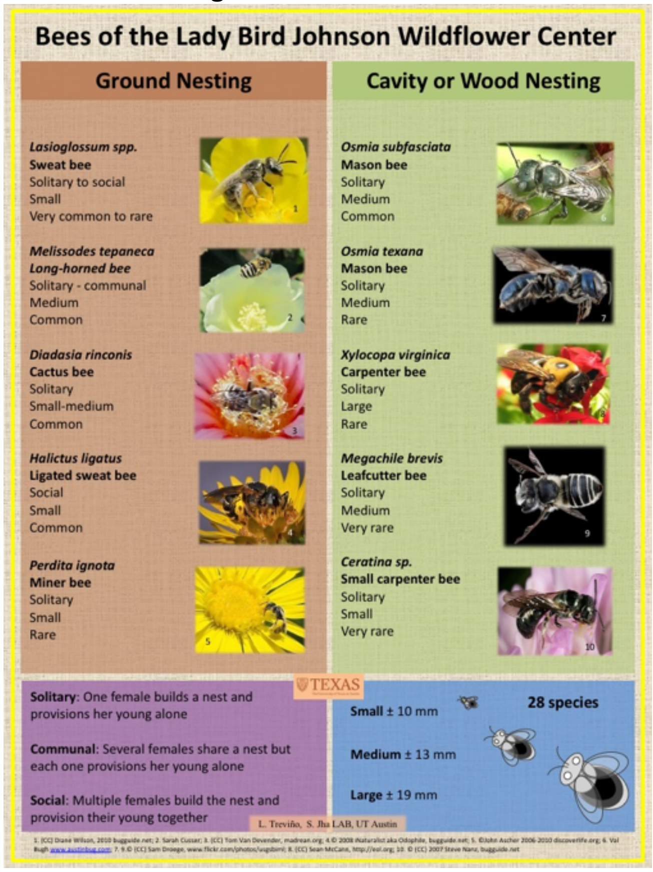

S2F. Best management practices (BMP) for native bee habitat conservation

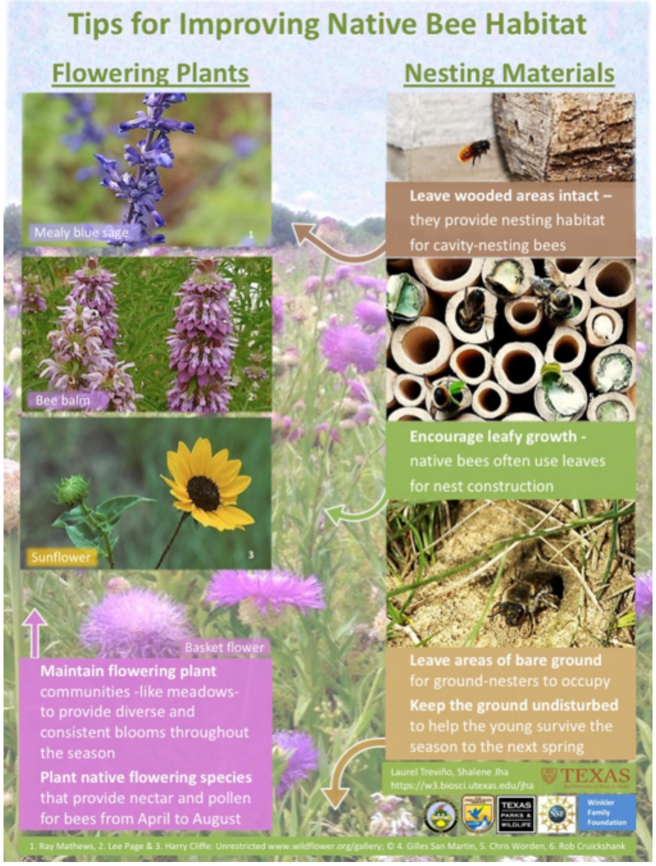

http://w3.biosci.utexas.edu/jha/wp-content/uploads//Native-Pollinator-Habitat.jpg

S2G. Pollinator Habitat Conservation Guidelines (2-sided pamphlet)

Threats to Pollinators

- Habitat loss
- Pesticides
- Diseases
- Climate change

How You Can Help

Provide food & water: Bees depend on nectar and pollen. Native bees prefer native plant pollen. Provide abundant and diverse native plants from spring through fall.

Conserve nest habitat: bare ground & cavities

Prevent: soil erosion & compaction

Reduce: pesticide use

Pollinator Habitat Management

Thanks to Texas Parks and Wildlife Department initiatives, landowners may now receive tax incentives by managing pollinator habitat. TPWD outlines Pollinator Habitat Conservation practices* to help landowners qualify for agriculture–wildlife tax valuation through their county appraisal district. A landowner must implement at least three habitat management practices such as pollinator surveys. The Jha Lab can help interpret data from pollinator surveys to inform landowners of best management practices (Landowners|Naturalists webpage).

http://w3.biosci.utexas.edu/jha/wp-content/uploads/Landowners|Naturalists.pdf

* http://tpwd.texas.gov/huntwild/wild/wildlife_diversity/nongame/native-pollinators/media/TPWD-Native-Pollinator-Management.pdf

5

Native Pollinators Thrive in Native Landscapes

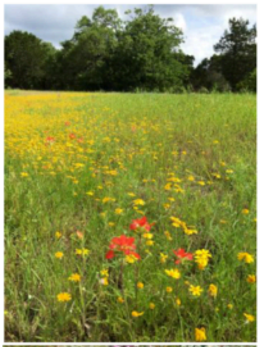

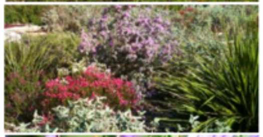

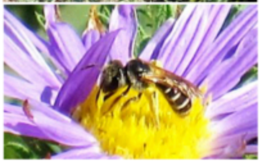

6

Pollinator Habitat Conservation

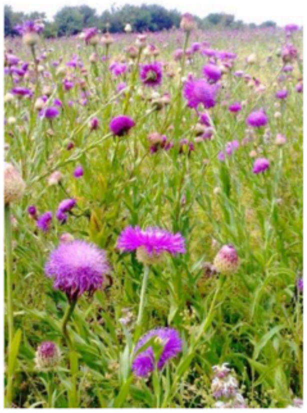

Laurel Treviño, M.S. & Shalene Jha, Ph.D.

The University of Texas at Austin
Department of Integrative Biology
College of Natural Sciences

1

Plants & Native Bees

~87% of flowering plants rely on animal pollinators to set fruit. There are 4,000 bee species in North America; 800+ of them in Texas alone. Native bees originated in and are adapted to local climate, soils, and native plants. Non-native species like the Western honeybee were introduced from Europe and became naturalized here.

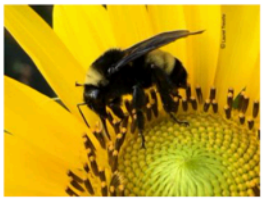

Bee-pollinated Crop Species

Almonds, apples & blueberries depend on bee pollination. Watermelons, melons and squash are often pollinated by native squash bees. Peppers, tomatoes and potatoes are often buzz pollinated by native bumble bees. Leafcutter bees pollinate alfalfa more efficiently than European/Western honeybees do.

Pollination services give us 1 in 3 food morsels!

~35% of U.S. crop species rely on insect pollination. Bees boost US crop production by ~$15 billion/year with ~$3 billion attributed to native bees. Studies in Texas show that insect mediated cross-pollination boosts cotton yield by 17%!

2

Native Bee Habitat

~75% of native bees are ground-nesters: digger bees, bumble bees, and sweat bees. If soil is deeply tilled, eroded or compacted their nests are destroyed. Leave bare ground for native bee nests!

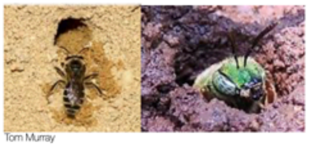

Tom Murray

~25% of native bees are cavity-nesters: carpenter bees, mason bees and leaf cutters make nests in soft wood, plant stems, rock crevices or snail shells. Nest sites are scarcer as native woodlands decrease. Leave snags, logs and thatch for nests!

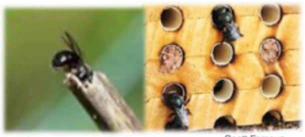

Alain C. Scott Famous

~90% of native bees are solitary. A female bee lays eggs on pollen balls she makes in her nest; larvae consume this bee bread to develop into pupa and then adults.

3

Hundreds of butterfly and bee species inhabit Central Texas

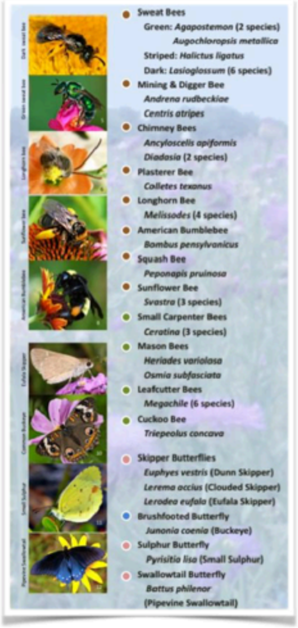

4

http://w3.biosci.utexas.edu/jha/wp-content/uploads//Pollinator-Habitat-Conservation.pdf

S2H. Central Texas prairie plants visited by native bees (2 pp. top 10 native plant families, genera, species)

Texas prairie plants for native bees

	Family	Common Name	Scientific Name
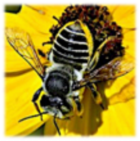 (CC) Bob Peterson Wikimedia Commons	*Asteraceae* Aster/daisy (most plant species, most bee species & abundance)	Fire wheel, Indian blanket<br>Texas thistle<br>Mexican hat<br>Golden wave	Gaillardia pulchella<br>Cirsium texanum<br>Ratibida columnifera<br>Coreopsis basalis
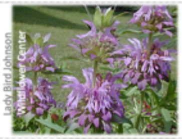 Lee Page, Lady Bird Johnson Wildflower Center	*Lamiaceae* Mints Sages	Beebalm/Wild bergamont<br>Blue/azure sage, pitcher sage<br>Drummond's skullcap<br>Mock pennyroyal/limoncillo	Monarda citriodora<br>Salvia azurea, S. texana<br>Scutelaria drummondii<br>Hedeoma drummondii
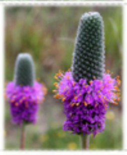 R.W. Smith, Lady Bird Johnson Wildflower Center	*Fabaceae* Legumes	Purple prairie clover<br>Golden, yellow prairie clovers<br>Nuttall's sensitive briar<br>Yellow puff sensitive briar	Dalea purpurea<br>D. aurea, D. nana<br>Mimosa nuttallii<br>Neptunia lutea
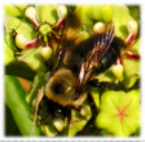 Laurel Treviño	*Asclepiadaceae* Milkweeds	Spider milkweed/antelope horns; green; green-flowered; pink/swamp milkweed (Not tropical *A. curassavica*)	Asclepias asperula, A. viridis, A. viridiflora, A. incarnate
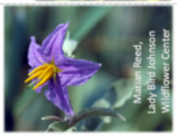 Marian Reed, Lady Bird Johnson Wildflower Center	*Solanaceae* Nightshades	Nightshade<br>Tomato weed<br>Buffalo bur nightshade	Solanum melongena<br>Solanum elaeagnifolium<br>Solanum rostratum

The University of Texas at Austin Department of Integrative Biology College of Natural Sciences

Laurel Treviño, S. Jha Lab

https://w3.biosci.utexas.edu/jha/about-native-bees - https://www.wildflower.org/collections/collection.php?collection=xerces_native

http://w3.biosci.utexas.edu/jha/wp-content/uploads//TX-Prairie-Plants-Native-Bees.pdf

S2I. Tips for establishing native plant landscapes for bee food and nesting resources (1 p.)

Native Landscapes for Native Bees

Pollinator habitat should provide resources for food and nesting.

Native bees prefer native plants over non-native plants,
except when invasive plants outcompete native congeners (same genus).

Bees love mints (beebalm, sage), legumes (bluebonnet, acacia), and
asters (daisy, sunflower). Build on a garden scaffold made of these plants.

Group colors and aromas in large patches or strips of the same plant species.

Plant diversity sustains bee diversity. A diverse garden, blooming spring through fall, will attract many species of bees and sustain a healthy pollinator community.

Relay planting periods for staggered blooming periods throughout the season.

Mix perennial (long-lived) and annual (short-lived) plants in a pollinator garden.

Laurel Treviño, M.S., Shalene Jha, Ph.D.

The University of Texas at Austin Department of Integrative Biology College of Natural Sciences

Scheme S3. Insect Labs A & B – view specimens on display with scopes & loupes

A. Distinguish Between Flower-Visiting Insects: Bees & Look-alikes

Flies: house fly, bombyliid fly (*Bombyliidae*), hoverfly (*Syrphidae*)

Wasp (Mexican honey wasp, flower wasp, paper wasp)

Hairy belly / Carder leafcutter bee (*Dianthidium* sp.)

Honey bee (*Apis mellifera, Apidae*)

B. Observe Native Bees in 4 of 6 North American/Texas Families

Bumble bee (*Bombus* sp., *Apidae*)

Large carpenter bee (*Xylocopa* sp., *Apidae*)

Tiny dark / Small carpenter bee (*Ceratina* sp., *Apidae*)

Hairy leg / Longhorn bee (*Melissodes* sp., *Apidae*)

Hairy leg / Sunflower / Cactus bee (*Diadasia* sp., *Apidae*)

Metallic, hairy belly / Mason (*Osmia* sp., *Megachilidae*)

Carder / leafcutter bee (*Dianthidium* sp., *Megachilidae*)

Hairy belly / Leafcutter bee (*Megachile* sp., *Megachilidae*)

Tiny dark sweat bee (*Lasioglossum* sp., *Halictidae*)

Striped sweat bee (*Halictus* sp., *Halictidae*)

Green sweat bee (*Agapostemon* sp., *Halictidae*)

Striped-abdomen, miner bee (*Perdita* sp., *Andrenidae*)

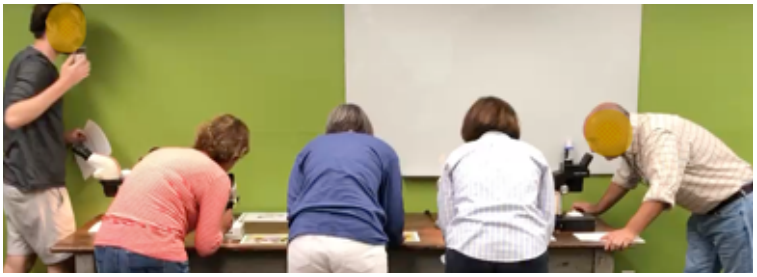

Scheme S4. Insect Labs - Example specimens of flower-visiting insects observed with scopes and loupes

FLIES (*Syrphidae & Bombyliidae*)

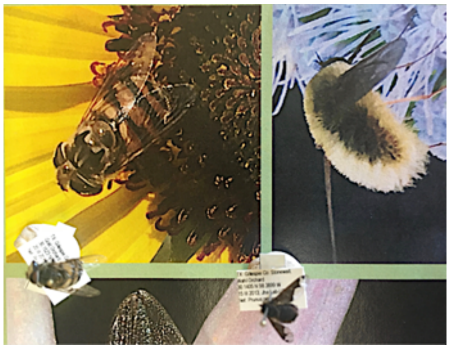

(*Syrphidae*)

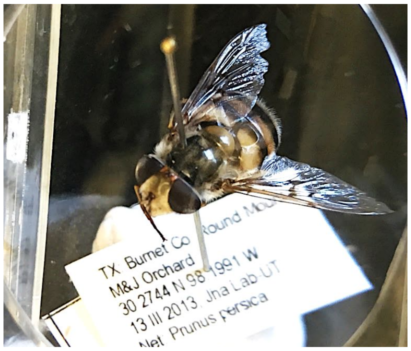

(*Tachinidae* house fly)

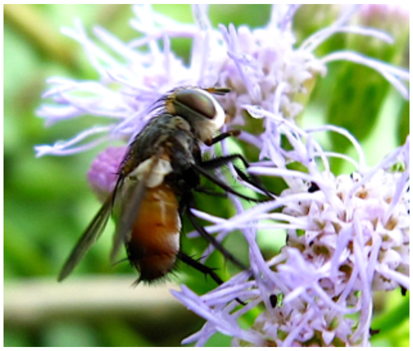

WASPS (Flower wasp)

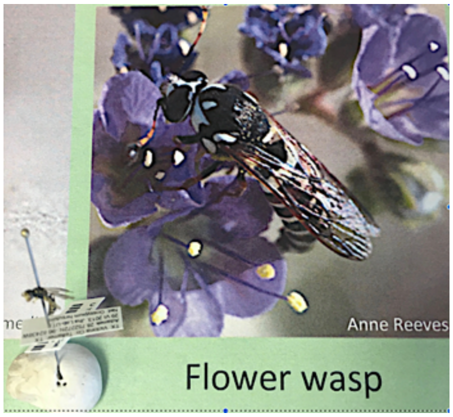

(Paper wasp, right)

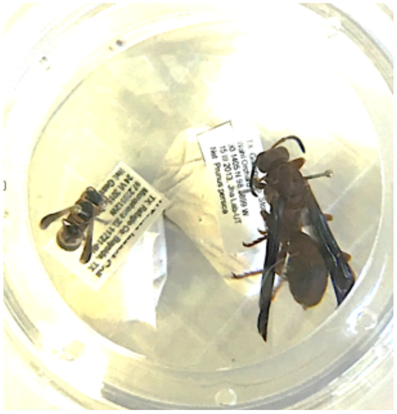

(Mexican honey wasp)

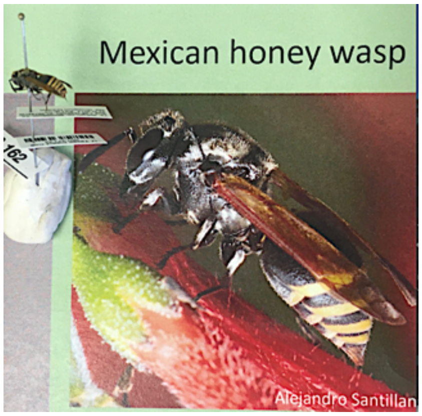

BEES (native leafcutter)

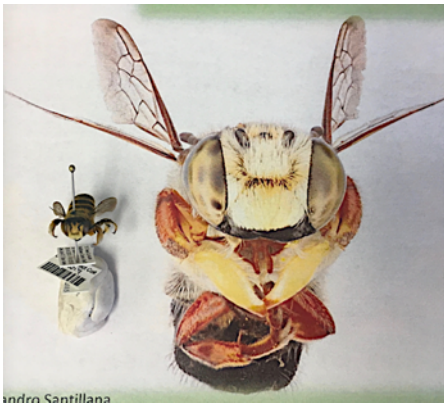

(native green sweat bees, m. f.)

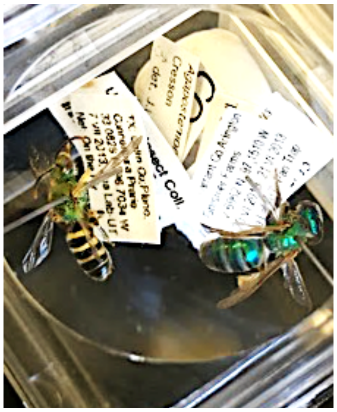

(Honey bee, *Apis mellifera*)

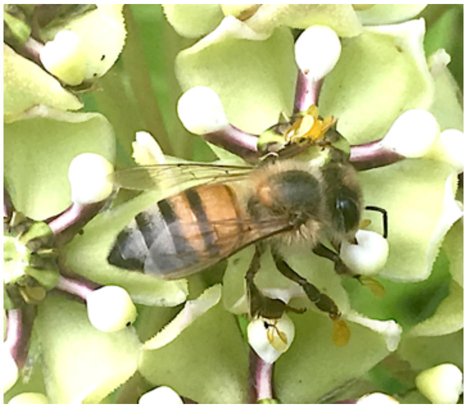

Scheme S5

5A. Fixed Route Survey - Instructions & Implementation

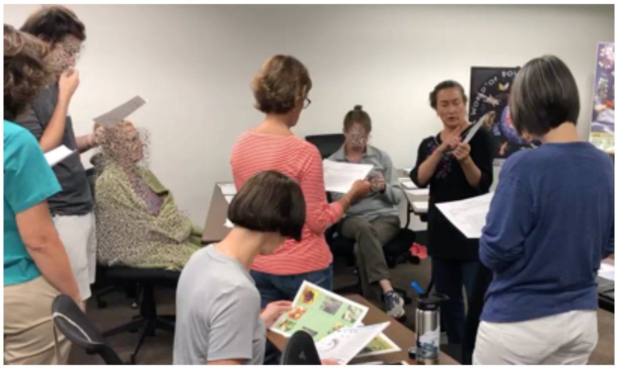

photo Carlos Torres Verdín

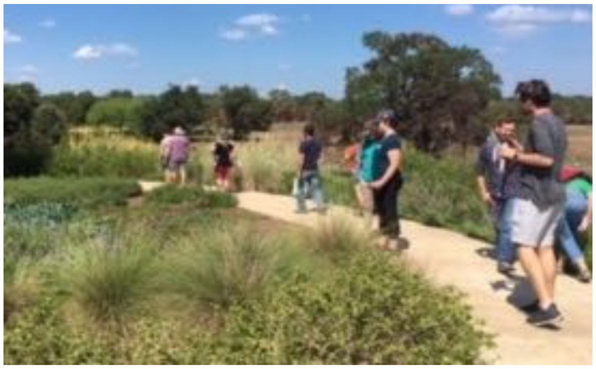

photo Shelly Engelman

Fixed Route Survey (80 m x 2 m), walk at a steady pace in one direction for 15 minutes
Identify & count insects on/in flowers only once, record observations on data sheet

5B. Data Sheet for Flower-visiting Insect Survey (Fixed Route)

Observer-Recorder Pair Names: ______________ _____________
Teamed with Expert (name): ______________
Team Color (circle one): RED, BLUE, YELLOW
Start time: __________ am/pm End time: __________ am/pm

INSECT	BEE GROUP	COUNT (卌 III)	SUM
BEE<br>Little or no hair on underside of abdomen<br><br>(pollen on leg hair or elsewhere)	Honey bee (*Apis mellifera*)		
	Bumble bee		
	Large carpenter bee		
	Hairy leg bee / longhorn		
	Green sweat bee		
	Striped sweat bee		
	Tiny dark bee		
	Striped abdomen - miner		
Brush of hair under abdomen carries pollen	Striped abdomen - plasterer		
	Striped abdomen - oil-collector		
	Hairy belly bee / leafcutter		
	Metallic hairy belly bee		
No hair	Cuckoo bee / parasitic bee		
	Unidentified native bee		
	(HB: honey bee, NB: native)	BEE COUNT	
NON-BEE	Flies		
	Wasps		
	Butterflies/Moths		
	Unidentified insect		
		NON-BEE INSECTS	
		ALL INSECTS	

http://w3.biosci.utexas.edu/jha/wp-content/uploads//Pollinator-Habitat-Surveys.pdf

Schema S6A. Life history and pollination topics covered in presentations & activities

Why are Native Bees Essential to Ecosystems?
Bee life history is linked to pollination behavior

Bee life history traits are tied to pollination functions. Pollinators & flowering plants co-evolved. Bee ephemeral life spans were in synch with flowering plant phenology. Bees are pollen/nectar herbivores. Pollination adaptations include collecting & carrying pollen loads on branched hair, females provisioning nests with pollen/nectar, and floral fidelity. Pollen preferences differ among bee species; while most are *polylectic*, few are *oligolectic* or *monolectic*.

How would you characterize native bees in Texas based on your acquired knowledge? ***Most North American native bees are solitary, ground-nesting, generalists.***

S6B. Life History & Behavior

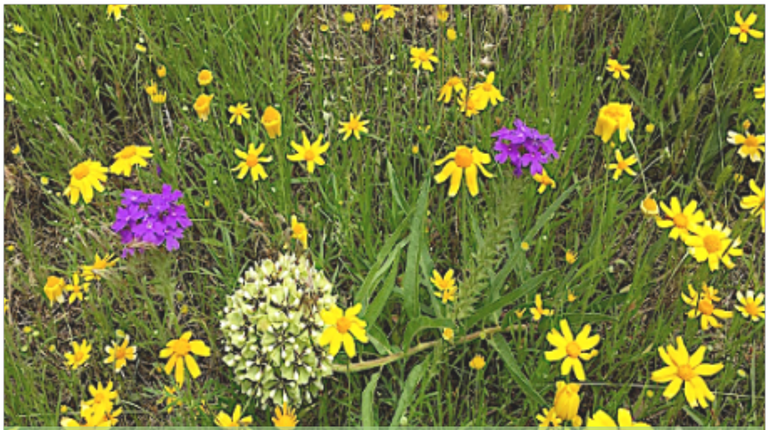

Life History & Behavior

- Foraging seasonality synchronous with flowering plant phenology
- Generalist herbivores eat pollen & nectar
- Ephemeral life span

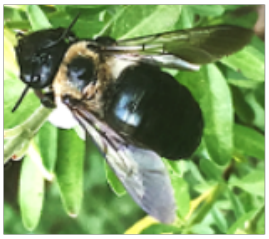

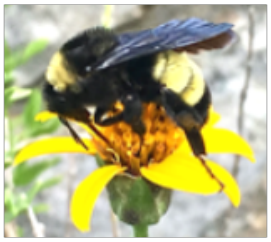

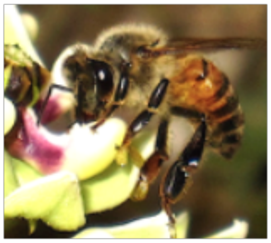

S6C. Pollination Adaptations

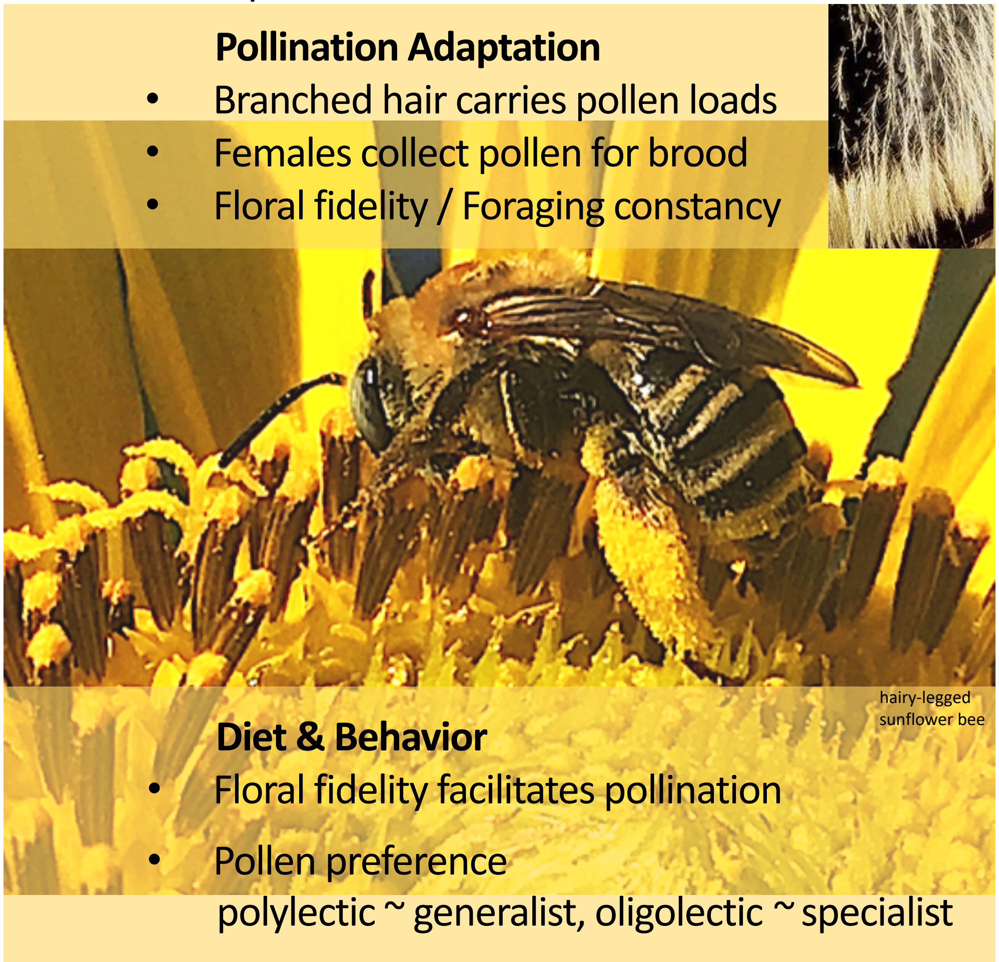

S6D. Nesting Behavior & Sociality

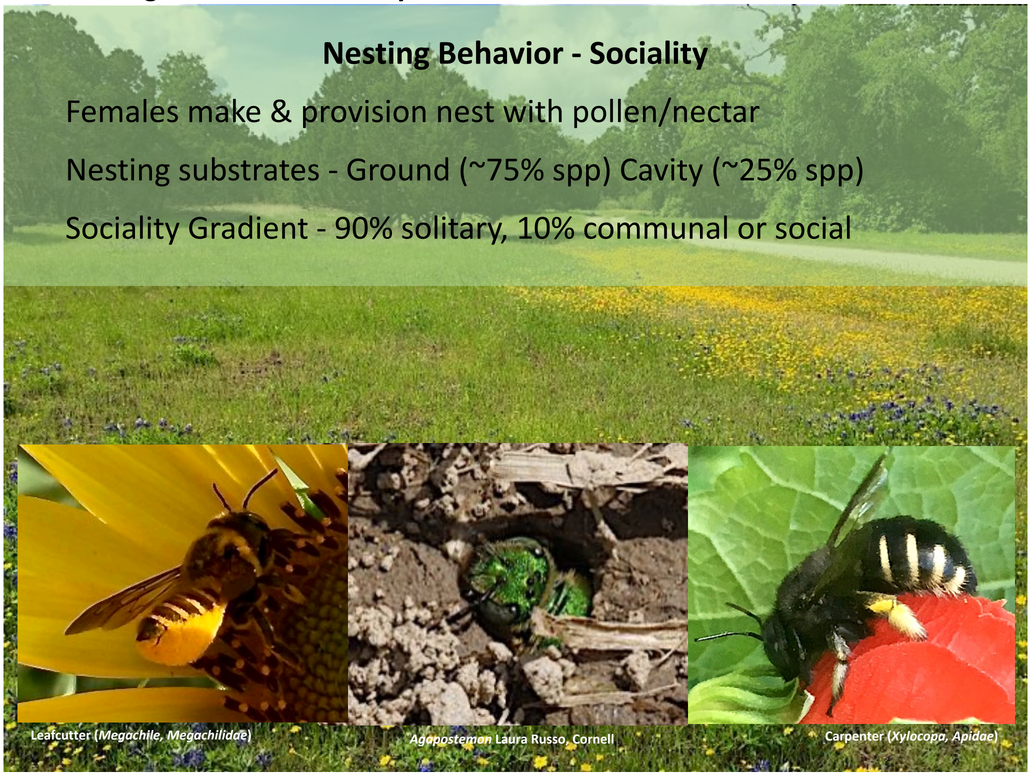

Scheme S7. Test questions #1-5 were projected on a screen for students' visual ID of flower-visiting insects

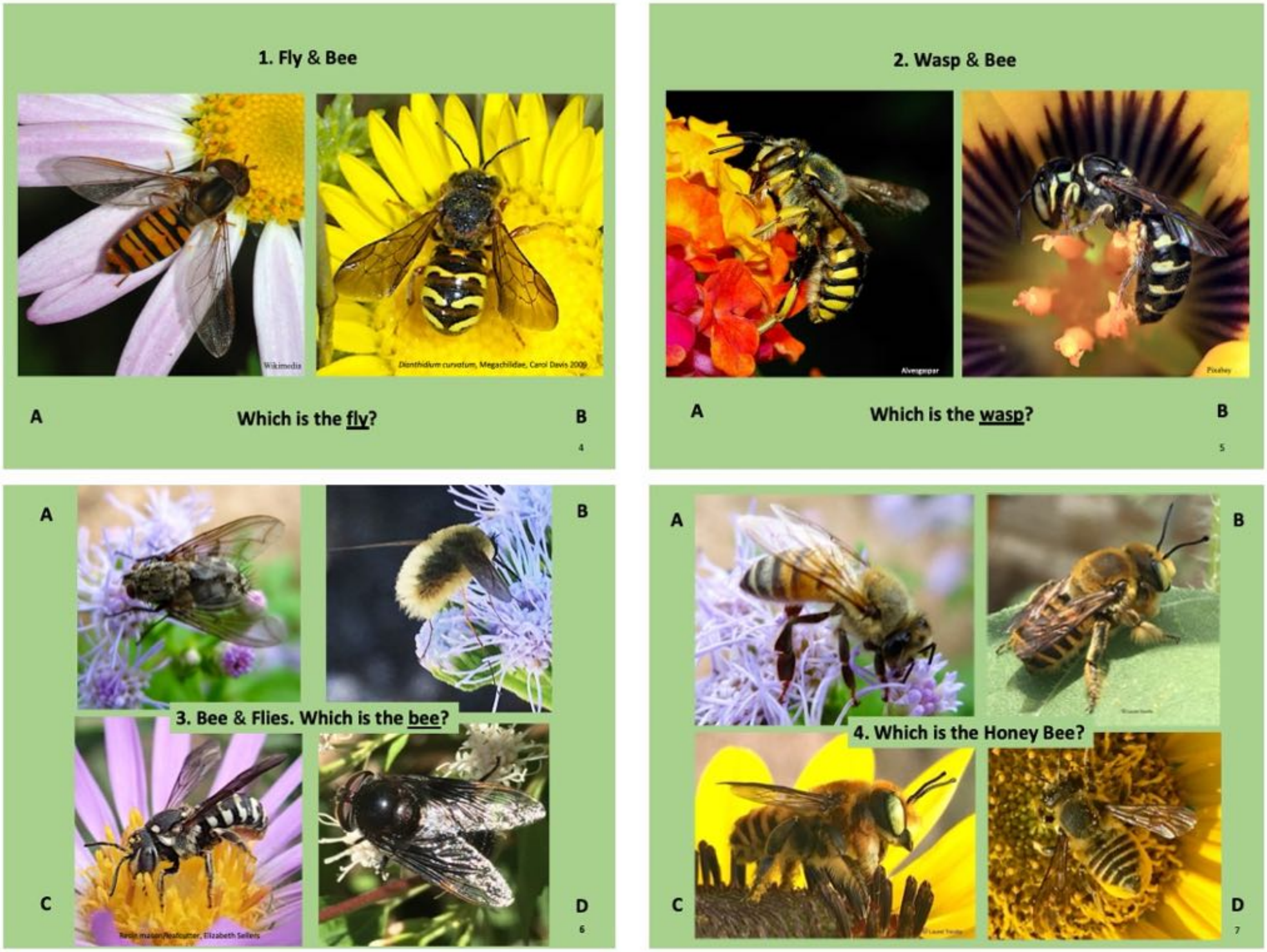

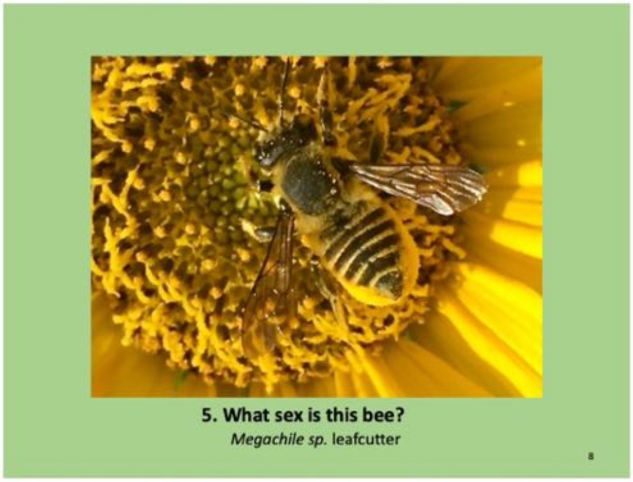

1. A. syrphid/hover fly
   B. *Dianthidium curvatum (Megachilidae)* bee
   resin mason leafcutter
2. A. *Anthidium florentinum (Megachilidae)* bee
   B. wasp
3. A. *Tachinidae* flesh fly
   B. *Bombyliidae* fly
   C. *Dianthidium curvatum* (*Megachilidae*)
   D. *Tabanidae* horse fly
4. A. *Apis mellifera (Apidae)* Western honey bee
   B, C. *Megachile fortis (Megachilidae)* male
   C. *Megachile* leafcutter bee (female); 5. *idem*

Note: Answer key (bottom right) for reference was not shown to students during tests in class

Scheme S8. Test questions #6-10 refer to topics on bee ecology content knowledge

"Native Bees of Texas" Course Quiz Answer Key Your Bee Name (2018) ____________________
Questions 1-5 refer to 5 slides of insects projected on the screen (previous page).

1. Fly & bee. Which is the fly? A, B

2. Wasp & bee. Which is the wasp? A, B

3. Bee & flies. Which is the bee? A, B, C, D

4. Four Bees. Which is the honey bee? A, B, C, D

5. What sex is this native leafcutter bee? A) male, B) female

6. Where do most native bee species nest (substrate in North America)?

A) cavities, B) ground, C) trees, D) all of these, E) I don't know

7. What features can help distinguish a bee from a fly or wasp in a garden?

A) 4 wings, B) fuzzy (branched) hair, C) flower constancy, D) all of these, E) I don't know

8. Why are bees important for ecosystem functions? Because they…

A) are food for wildlife, B) pollinate most plants, C) help produce fruits & seeds, D) all, E) I don't know

9. What pollination services do bees provide for us?

A) improve fruit quality, B) boost fruit yield, C) pollinate most of our fruits, D) all, E) I don't know

10. Why are native bee populations declining?

A) they aren't multiplying, B) habitat loss, C) they reached carrying capacity, D) all, E) I don't know

What occupation or hobby best describes you? Please, circle or state (include retired/current).

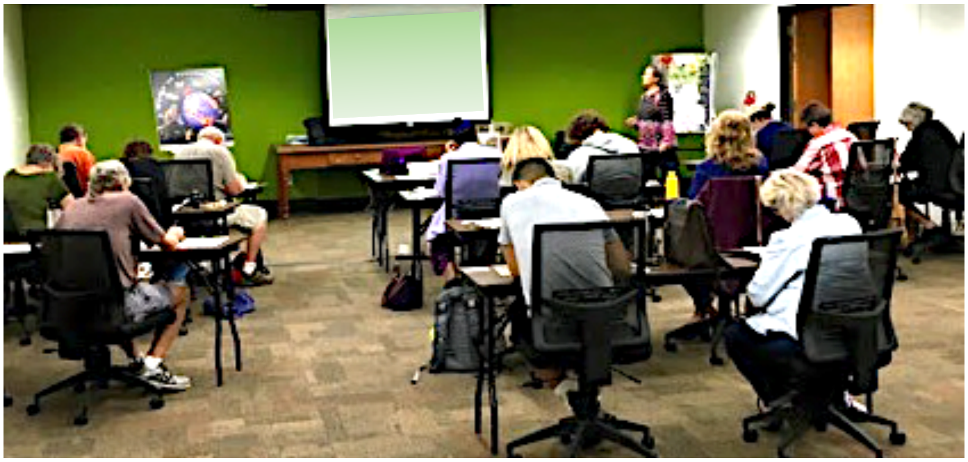

Photo Carlos Torres Verdín

Scheme S9. Feedback Form: Overall & specific knowledge and open-ended feedback (top) 2018 & 2019
Participant retrospective self-rating of perceived knowledge gains (bottom) – 2019 only

Feedback Form - Native Bees of Texas Course

On a scale of 1-5, please rate this class based on the following criteria:

How informative it was / How useful the knowledge or skills will be / How engaged were you as a participant?

Not informative	1	2	3	4	5	Informative
Not useful	1	2	3	4	5	Useful
Not engaged	1	2	3	4	5	Engaged

As a result of today's workshop, I am better able to …

Understand the importance of native bees in ecosystems and agroecosystems

Strongly disagree	Disagree	Neutral	Agree	Strongly agree
1	2	3	4	5

Distinguish native bees from other flower visiting insects that look like them

Strongly disagree	Disagree	Neutral	Agree	Strongly agree
1	2	3	4	5

Identify common native bees of Central Texas with the help of the basic guides we used in this class

Strongly disagree	Disagree	Neutral	Agree	Strongly agree
1	2	3	4	5

Use this foundation of bee family taxonomy to learn more about the huge diversity of native bees on my own

Strongly disagree	Disagree	Neutral	Agree	Strongly agree
1	2	3	4	5

Use best management practices to conserve native bee habitat (based on nest habits, life cycles, plants)

Strongly disagree	Disagree	Neutral	Agree	Strongly agree
1	2	3	4	5

Choose the best combinations of native prairie plants to make a native bee garden in Central Texas

Strongly disagree	Disagree	Neutral	Agree	Strongly agree
1	2	3	4	5

Describe how you intend to use skills/knowledge that you learned in today's sessions in your practice/home

The best part(s) of this event was/were ____

This event could be improved by ____

**PRE**<br>**BEFORE** participating in this workshop, I would rate my knowledge of native bees as:	**POST**<br>**NOW**, I would rate my knowledge of native bees as:
○ Poor<br>○ Fair<br>○ Good<br>○ Very Good<br>○ Excellent	○ Poor<br>○ Fair<br>○ Good<br>○ Very Good<br>○ Excellent

Laurel Treviño, M.S.; Shalene Jha, Ph.D. "Jha Lab", Integrative Biology Dept.; Shelley Engelman, Ph.D., Adrienne Loftus and Haley Barnes, Texas Institute for Discovery Education in Science STEM Program for Research & Evaluation; College of Natural Sciences, The University of Texas at Austin
