# Supplementary material for: The Native Bees of Texas: Evaluating the Benefits of a Public Engagement Course
Source: Insects. 2021 Aug 5;12(8):702. doi: 10.3390/insects12080702 (PMC8396608; doi:10.3390/insects12080702)
Supplement: Supplementary file 1 [file insects-12-00702-s001.zip › Supplementary-Tables_Native-Bees-Texas.pdf]

Tables - *The Native Bees of Texas: Evaluating the benefits of a public engagement course*

Table S1. Demographics - anonymous background information (2019, n=21)

Gender	DATA	Age Range	DATA
○ Female<br>○ Male<br>○ Other (please specify)	F 14 (67%)<br>M 7 (33%)	○ Under 18<br>○ 18-24<br>○ 25-34<br>○ 35-44<br>○ 45-54<br>○ 55+	18-24: 1 (5%)<br>35-44: 5 (23%)<br>45-54: 6 (29%)<br>55+: 9 (43%)
Ethnicity		Highest degree or school level	
○ White<br>○ Hispanic or Latinx<br>○ Black or African American<br>○ Native American/Indian<br>○ Asian/ Pacific Islander<br>○ Multiracial<br>○ Other (please specify)	White 100%	○ Less than a high school diploma<br>○ High school degree/equivalent (e.g., GED)<br>○ Associate degree (e.g., AA, AS)<br>○ Bachelor's degree (e.g., BA, BS)<br>○ Master's degree (e.g., MA, MS, MEd)<br>○ Professional degree (e.g., MD, DDS, DVM)<br>○ Doctorate (e.g., PhD, EdD)<br>○ Other (please specify)	HS 1 (4.8%)<br>B 10 (47.6%)<br>M 9 (42.8%)<br>D 1 (4.8%)
Employment status		Current or former occupations	(2018+2019)
○ Employed full time (40 h)<br>○ Employed part time (20 h)<br>○ Student<br>○ Retired<br>○ Homemaker<br>○ Self-employed	FT 13 (68%)<br>PT 1 (5%)<br>Ret. 4 (21%)<br>Stu. 1 (5%)	11 Naturalist<br>6 Gardener<br>4 Education (Teacher/Professor)<br>1 Environmental Education<br>1 Biology<br>1 Farm / Ranch owner / manager<br>4 Real estate<br>3 Student (college)<br>3 Photography<br>2 Environmental Engineering<br>3 Government - training manager<br>1 Immigration<br>1 NGO worker<br>1 IT project manager<br>1 Physical therapy<br>1 Homemaker	Reason / motivation to take course<br><br>curiosity (7), acquiring practical knowledge (10), receiving training credit Master Naturalists (10) in 2019
Household income/year	N/A	Primary Residence (ZIP code)	(15) 786- -<br>(4) 787- -<br>(1) 768- -<br>(1) 781- -

Table S2. Test Results (Mean % test scores per question across participants, *p<0.05, **p<0.01) (A. 2018, B. 2019)

Test - % Correct Responses Pre & Post-Course	A. 2018 (n = 11)					B. 2019 (n = 19)				
Standardized Test Questions	Pre	SE	Post	SE	p-value	Pre	SE	Post	SE	p-value
Fly & bee. Which is the fly?	73%	0.14	91%	0.09	0.303	100%	0.00	100%	0.00	1.000
Wasp & bee. Which is the wasp?	73%	0.14	73%	0.14	1.000	84%	0.09	68%	0.11	0.187
Bee & flies. Which is the bee?	36%	0.15	73%	0.14	0.102	63%	0.11	79%	0.10	0.331
Bees. Which is the honey bee?	55%	0.16	82%	0.12	0.193	42%	0.12	68%	0.11	0.056
What sex is this leafcutter bee?	45%	0.16	91%	0.09	0.028*	42%	0.12	84%	0.09	0.007**
What features help distinguish bees, flies, wasps?	55%	0.16	91%	0.09	0.068	53%	0.12	89%	0.07	0.005**
Where do most native bees nest?	64%	0.15	100%	0.00	0.035*	21%	0.10	95%	0.05	0.000**
Why are bees important for ecosystem function?	55%	0.16	73%	0.14	0.408	74%	0.10	100%	0.00	0.021*
What pollination services do bees provide us?	55%	0.16	100%	0.00	0.015*	84%	0.09	100%	0.00	0.083
Why are native bee populations declining?	91%	0.09	100%	0.00	0.363	74%	0.10	89%	0.07	0.083
**Overall Mean (n=10)**	**60%**	**0.05**	**87%**	**0.03**	**0.009****	**64%**	**0.04**	**87%**	**0.02**	**0.000****

Table S3. Test Results (Differences by category: ID skills & ecological knowledge, sorted from large to small)

Test	Topic Category	A. 2018 (n=11)			B. 2019 (n=19)		
		Pre	Post	Diff	Pre	Post	Diff
ID Skills	Bee's Sex	45%	91%	46%	42%	84%	42%
	Bee or Fly	36%	73%	37%	63%	79%	16%
	Honey v. Native Bee	55%	82%	27%	42%	68%	26%
	Fly or Bee	73%	91%	18%	100%	100%	0%
	Wasp or Bee	73%	73%	0%	84%	68%	-16%
	**Overall Mean (n=5)**	**56%**	**82%**	**25.6%**	**66%**	**80%**	**13.6%**
	SE	0.08	0.04	7.93	0.12	0.06	10.05
Eco Know	Nesting Behavior	64%	100%	36%	21%	95%	74%
	Bee Features	55%	91%	36%	53%	89%	36%
	Pollination Services	55%	100%	45%	84%	100%	16%
	Ecosystem Functions	55%	73%	18%	74%	100%	26%
	Bee Population Declines	91%	100%	9%	74%	89%	15%
	**Overall Mean (n=5)**	**64%**	**93%**	**28.8%**	**61%**	**95%**	**33.4%**
	SE	0.07	0.05	6.60	0.11	0.03	10.82
	**Overall Mean (N=10)**	**60%**	**87%**	**27.2%**	**64%**	**87%**	**23.5%**
	SE	-	-	4.90	-	-	7.72

Table S4. Insect Survey (2019)

	Abundance	Experts				Students			
		Sum	Prop	Mean	SE	Sum	Prop	Mean	SE
Insect Groups	Native bees	114	0.61	38.00	8.97	72	0.58	24.00	5.01
	Honey bees (*Apis mellifera*)	47	0.25	15.67	7.32	26	0.22	8.67	4.49
	Butterflies	14	0.07	4.67	1.97	11	0.09	3.67	3.00
	Flies	7	0.04	2.33	0.98	7	0.06	2.33	1.51
	Wasps	6	0.03	2.00	0.82	7	0.06	2.33	1.09
Bee Groups	Honey bees (*Apis mellifera*)	47	0.29	15.67	7.32	26	0.27	8.67	4.49
	Striped hairy belly leafcutter (*Megachile* sp.)	42	0.26	14.00	3.22	15	0.16	5.00	5.01
	Large carpenter bee (*Xylocopa* 2 spp)	25	0.16	8.33	1.46	6	0.06	2.00	1.53
	Hairy leg (*Diadasia sp., Melissodes sp., Centris sp.*)	23	0.14	7.67	2.40	3	0.03	1.00	0.58
	Bumble bee (*Bombus pensylvanica*)	22	0.14	7.33	2.40	42	0.44	14.00	3.01
	Tiny dark bee (*Lasioglossum* sp., *Ceratina* sp.)	1	0.01	0.33	0.34	1	0.01	0.33	0.34
	Cuckoo/parasitic bee (*Coelioxys* sp.)	1	0.01	0.33	0.34	0	0.00	0.00	0.00
	Striped abdomen plasterer (NA)	0	0.00	0.00	0.00	3	0.03	1.00	1.00
	Green bee (NA)	0	0.00	0.00	0.00	2	0.02	0.67	0.66

Table S5. General Perceived Knowledge: participant retrospective self-rating of overall perceived knowledge gains
Mean ratings, on a 5-point Likert scale (1-poor to 5-excellent) per question across all participants, 2019 (n=20)
A Mann-Whitney U test assessed statistically significant differences from pre to post ratings (t-test p-value **p<0.01)
Using a retrospective self-rating, "I would rate my knowledge of native bees as..."

Retrospective Self-ratings	n	Mean	SE	p-value	Poor (1)	Fair (2)	Good (3)	Very Good (4)	Excellent (5)
Before/Pre	20	1.65	0.21	0.000**	60%	20%	15%	5%	0%
Now/Post	20	3.35	0.18		0%	10%	55%	25%	10%

Table S6. Specific Perceived Knowledge: participant ratings of knowledge
Mean ratings of six specific topics, on a 5-point Likert scale (1, Strongly disagree to 5, Strongly agree) per question across all participants, A. 2018 (n=12), B. 2019 (n=19)
"As a result of this course, I'm better able to ..."

	A. 2018 (n = 12)							B. 2019 (n = 19)						
Specific Topics	Mean	SE	(1)	(2)	(3)	(4)	(5)	Mean	SE	(1)	(2)	(3)	(4)	(5)
Ecosystem Services	4.75	0.13	0%	0%	0%	25%	75%	4.58	0.12	0%	0%	0%	42%	58%
Distinguish bee insects	4.50	0.15	0%	0%	0%	50%	50%	4.26	0.13	0%	0%	5%	63%	32%
Identify native bees	4.33	0.14	0%	0%	0%	67%	33%	4.00	0.15	0%	0%	21%	58%	21%
Taxonomy Diversity	4.33	0.14	0%	0%	0%	67%	33%	4.37	0.16	0%	0%	11%	42%	47%
Conservation	4.50	0.15	0%	0%	0%	50%	50%	4.74	0.10	0%	0%	0%	26%	74%
Native bee plants	4.25	0.18	0%	0%	8%	58%	33%	4.53	0.14	0%	0%	5%	37%	58%
Overall Mean (n=6)	4.44	0.07	-	-	-	-	-	4.41	0.11	-	-	-	-	-

Table S7. Specific Topic Categories: Visual ID Skills and Ecological/conservation Knowledge (from Table S6 above)

ID Skills	A. 2018	B. 2019	Eco/Conserve Know	A. 2018	B. 2019
Distinguish insects	4.50	4.26	Ecosystem services	4.75	4.58
Identify native bees	4.33	4.00	Conservation	4.50	4.74
Taxonomy Diversity	4.33	4.37	Native bee plants	4.25	4.53
Mean (n=3)	4.39	4.21	Mean (n=3)	4.50	4.62
SE	0.06	0.11	SE	0.14	0.06

Table S8. Overall Course Ratings. "On a scale of 1 to 5, the class was..."
Mean ratings, on a 5-point Semantic Differential Scale (1, Not at all to 5, Very much), expressed as percent of each criterion

Criteria	A. 2018 (n = 11)							B. 2019 (n = 19)						
"It was..."	Mean	SE	(1)	(2)	(3)	(4)	(5)	Mean	SE	(1)	(2)	(3)	(4)	(5)
Informative	5.00	0.0	0%	0%	0%	0%	100%	4.95	0.05	0%	0%	0%	5%	95%
Useful	5.00	0.0	0%	0%	0%	0%	100%	4.74	0.13	0%	0%	5%	16%	79%
Engaging	5.00	0.0	0%	0%	0%	0%	100%	4.26	0.17	0%	0%	16%	42%	42%
Mean (n=3)	5.00	0.0	-	-	-	-	-	4.65	0.20	-	-	-	-	-

Table S9. Applications of Acquired Knowledge. 2018 (n=11), 2019 (n=19)

Category	2018, participants reported they intended to...	2019, participants reported they intended to...
Gardening / Land Management	Increase diversity of bees in my backyard<br>Add bare ground<br>Keep more leaves on ground as mulch<br>Reduce use of insecticides<br>Grow pollinator garden<br>Plant native plants<br>Plant more native plants<br>Plant more bee-specific plants<br>Convert lawn to native Texas wildflower meadow	Grow as much as I can from an apartment<br>Lawn and garden care and development<br>Build habitat / Home landscaping<br>Convert yard / Create to pollinator gardens<br>Plant flowers / bee-friendly plants<br>Increase flowers for native meadows / prairies<br>Increase bee-friendly wildflowers on ranch<br>Continue to establish native grasses/range science<br>Land management for pollinators<br>Identify what I have & plan to improve habitat<br>Document, increase number/variety bees on ranch
Education / Projects	Teach about native bee populations and behavior<br>Teach others & improve habitat<br>Project for UT course “Sustaining a Planet”	Share knowledge / ID photo project<br>Present basic info to clubs master gardeners<br>Continue work with Texas Master Naturalists<br>Teach Master Gardeners native bee habitat
SUMMARY	82% apply acquired know/skills of native bee ecology/conservation in their garden or land management<br>26% apply acquired know/skills of native bee ecology/conservation to educational projects<br>18% expressed learning about solitary bee behavior helped understand & manage for native bees<br>18% highlighted importance of learning diversity & ID to distinguish between flower-visiting insects<br>9.5% underlined importance/application of improving native bee habitat for pollinator conservation	

Table S10. Effective Course Aspects

Category	2018 The best part of the course was…	2019 The best part of the course was…
Content Knowledge & Applicability	Identification & conservation of native bees<br>I didn't know that many bees were ground-nesters<br>Knowing how many bees nest in the ground, makes a huge difference in how I will garden in the future	All the good info./ All of it / Everything; it was great<br>I found I knew nothing before learning about bees<br>Knowing about all the types and sizes of bees<br>So much! Learning to distinguish bees/flies/wasps<br>Learning bee behavior / how to provide habitat<br>Understand/ realize native bees are mostly solitary<br>[You] don't need honey bees to improve ecosystems
Format / Instruction	All parts were extremely useful<br>Nice mixture of lecture, lab, and field time<br>Presentation style with photos on screen<br>Learning from an expert<br>I thought the class was great	Both, lectures & labs complemented learning process<br>Bee examples<br>Learning how to better teach this topic & how to provide habitat<br>Excellent instructor
Hands-on learning	Going outside<br>Exploring the Wildflower Center gardens<br>Being in the Wildflower Center<br>Learning more about native life	I thought it was great seeing actual bees under scopes<br>Observations with scopes<br>Bee labs<br>Seeing actual bees
Resources	Hand-outs [printed material]<br>Bee photos [projected] on screen	Hand-outs<br>Slides of bees and Bee examples
SUMMARY	52% highlighted usefulness/applicability, 36% underlined behavior/ecology applications to conservation<br>42% appreciated excellent expert instructor (14% appreciated the presentation style & course format)<br>28% highlighted the hands-on learning, directly observing insects with scopes and in native gardens<br>14% found slides & printed educational material to be useful resources	

Table S11. Suggested Course Improvements

Category	2018 The course could be improved by …	2019 The course could be improved by …
Duration & Pace	Longer time in the gardens<br>I wish the course were longer or a multi-segmented [course]<br>Increase time by a couple of hours	Slower pace<br>Longer time for involvement<br>More field time
Instruction	1 student did not understand the scatterplot of bee abundance v. ground cover	Less detail on [bee] metamorphosis<br>Label & identify bees at scopes [better]
Topics	Nothing that could have been done by the instructor<br>Suggest sharing more between participants<br>Talk more about plant identification	
SUMMARY	21% suggested a longer workshop, more time in gardens, sharing more information among participants	
