# Supplementary material for: The Native Bees of Texas: Evaluating the Benefits of a Public Engagement Course
Source: Insects. 2021 Aug 5;12(8):702. doi: 10.3390/insects12080702 (PMC8396608; doi:10.3390/insects12080702)
Supplement: Supplementary file 1 [file insects-12-00702-s001.zip › Native Bees of Texas Data Available Public Archive.zip!/Native Bees of Texas Course_Insect Survey_All Data_2019.pdf]

3 TEAMS (RED, BLUE, YELLOW) 2 STUDENTS AND 1 EXPERT FIXED ROUTE SURVEY FLOWER VISITING INSECTS

INSECTS - expert species, student genera	FAMILY	Expert	Students	Expert	Students	Expert	Students
Honey bee (Apis mellifera)	Apidae	6	15	11	0	30	11
Bumble bee (Bombus pensylvanica)	Apidae	6	17	4	17	12	8
Large carpenter bee (Xylocopa virginica)	Apidae	5	1	7	0	11	5
Large carpenter bee (Xylocopa micans)	Apidae	1	0	1	0	0	0
Hairy leg/Long-horned (Diadasia enavata)	Apidae	1	1	3	0	0	0
Hairy leg/Long-horned (Melissodes sp.)	Apidae	7	0	0	0	3	2
Hairy leg/Long-horned (Centris atripes)	Apidae	1	0	0	0	8	0
Green bee (possible green sweat bee)	Halictidae	0	0	0	2	0	0
Tiny dark bee (Lasioglossum/Ceratina)	Hal. Apid.	0	0	0	0	1	1
Striped abdomen plasterer	Colletidae	0	0	0	0	0	3
Striped hairy belly leafcutter (Megachile)	Megachilidae	5	0	8	0	19	15
Megachile exile	Megachilidae	7	0	0	0	0	0
Anthidiellum sp.	Megachilidae	3	0	0	0	0	0
Cuckoo/parasitic bee (Coelioxys sp)	-	1	0	0	0	0	0
Unidentified native bees (Megachile?)		0	9	0	88	0	0
Fly bombyliid	Bombyliidae	0	6	4	1	3	0
Wasp Mexican honey	Vespidae	1	1	1	5	4	1
Butterfly skipper	Hesperiidae	8	11	6	0	0	0

GROUPED (EXPERT'S) SPECIES FROM TABLE ABOVE INTO (STUDENT'S) BEE GROUPS (PARATAXONOMIC UNITS)

INSECT Genera or para-taxonomic groups (grouped species from table above)	FAMILY	Red Expert	Red Students	Blue Expert	Blue Students	Yellow Expert	Yellow Students
Honey bee (Apis mellifera)	Apidae	6	15	11	0	30	11
Bumble bee	Apidae	6	17	4	17	12	8
Large carpenter bee (2 spp)	Apidae	6	1	8	0	11	5
Hairy leg/Longhorn (3 spp)	Apidae	9	1	3	0	11	2
Green sweat bee	Halictidae	0	0	0	2	0	0
Tiny dark bee	Hal. Apid.	0	0	0	0	1	1
Striped abdomen plasterer	Colletidae	0	0	0	0	0	3
Striped hairy belly leafcutter (2 spp)	Megachilidae	15	0	8	0	19	15
Cuckoo/parasitic bee	-	1	0	0	0	0	0
Unidentified native bee	-	0	9	0	88	0	0
Fly bombyliid	Bombyliidae	0	6	4	1	3	0
Wasp (Mexican honey, Brachystaga mellifera)	Vespidae	1	1	1	5	4	1
Butterfly skipper	Hesperiidae	8	11	6	0	0	0

SUMMARY TABLE – FAMILY & GENERA OBSERVED BY EXPERTS, 3 TEAMS
(Unidentified bees were dropped because there was no expert identification/verification or corresponding data)

FAMILY	Insect Genera (Experts)	R Expert	B Expert	Y Expert	SUM	MEAN
Apidae	Honey bee (Apis mellifera)	6	11	30	47	15.67
Megachilidae	Striped hairy belly leafcutter (Megachile)	15	8	19	42	14.00
Apidae	Large carpenter bee (Xylocopa 2)	6	8	11	25	8.33
Apidae	Hairy leg/Longhorn (3 spp)	9	3	11	23	7.67
Apidae	Bumble bee (Bombus)	6	4	12	22	7.33
Hal. Apid.	Tiny dark bee (Lasioglossum/Ceratina)	0	0	1	1	0.33
-	Cuckoo/parasitic bee (Coelioxys)	1	0	0	1	0.33
Hesperidae	Butterfly (Skipper)	8	6	0	14	4.67
Bombyliidae	Fly (Bombyliidae)	0	4	3	7	2.33
Vespidae	Wasp (Mexican honey) Brachystaga	1	1	4	6	2.00
	ALL BEE COUNTS	43	34	84	161	53.67
	NATIVE BEE COUNTS	37	23	54	114	38.00
	NON-BEE INSECT / COUNTS	9	11	7	9	
	ALL INSECTS				188	

SUMMARY TABLE – FAMILY & GENERA OBSERVED BY STUDENTS, 3 TEAMS

FAMILY	Insect Genera counts (Students)	R Students	B Students	Y Students	MEAN
1	Honey bee	15	0	11	8.67
2	Bumble bee	17	17	8	14
3	Large carpenter bee	1	0	5	2
4	Hairy leg/Longhorn	1	0	2	1
5	Green sweat bee	0	2	0	0.67
6	Tiny dark bee	0	0	1	0.33
7	Striped abdomen plasterer	0	0	3	1
8	Striped hairy belly leafcutter	0	0	15	5
9	UnID native bee (leafcutter Gregg's dalea)	9	88	0	32.33
	ALL BEE COUNTS	43	107	45	65
	NATIVE BEE COUNTS	28	107	34	56.33
1	Fly	6	1	0	2.33
2	Wasp	1	5	1	2.33
3	Butterfly	11	0	0	3.67
	NON-BEE INSECT COUNTS	18	6	1	8.33

SUMMARY TABLE – BEE GROUPS OBSERVED BY EXPERTS AND STUDENTS

BEE GROUPS	EXPERT					STUDENT				
	SUM	PROP.	MEAN	SD	SE	SUM	PROP.	MEAN	SD	SE
Honey bee (Apis mellifera)	47	0.29	15.67	12.66	7.32	26	0.27	8.67	7.77	4.49
Striped hairy belly leafcutter (Megachile)	42	0.26	14.00	5.57	3.22	15	0.16	5.00	8.66	5.01
Large carpenter bee (Xylocopa 2 spp)	25	0.16	8.33	2.52	1.46	6	0.06	2.00	2.65	1.53
Hairy leg/Long-horn (3 spp)	23	0.14	7.67	4.16	2.40	3	0.03	1.00	1.00	0.58
Bumble bee (Bombus)	22	0.14	7.33	4.16	2.40	42	0.44	14.00	5.20	3.01
Tiny dark bee (Lasioglossum/Ceratina)	1	0.01	0.33	0.58	0.34	1	0.01	0.33	0.58	0.34
Cuckoo/parasitic bee (Coelioxys)	1	0.01	0.33	0.58	0.34	0	0.00	0.00	0.00	0.00
Striped abdomen plasterer	0	0.00	0.00	0.00	0.00	3	0.03	1.00	1.73	1.00
Green sweat bee	0	0.00	0.00	0.00	0.00	2	0.02	0.67	1.15	0.66
Native bee COUNT / Proportion	114	0.71				72	0.73			
TOTAL BEE COUNT	161	1				98	1			

INSECT GROUPS	EXPERTS					STUDENTS				
	SUM	PROP.	MEAN	SD	SE	SUM	PROP.	MEAN	SD	SE
Native bee	114	0.61	38.00	15.52	8.97	72	0.58	24.00	8.66	5.01
Honey bee	47	0.25	15.67	12.66	7.32	26	0.22	8.67	7.77	4.49
Butterfly	14	0.07	4.67	3.40	1.97	11	0.09	3.67	5.19	3.00
Fly	7	0.04	2.33	1.70	0.98	7	0.06	2.33	2.62	1.51
Wasp	6	0.03	2.00	1.41	0.82	7	0.06	2.33	1.89	1.09

NOTE
I calculated means w different (n = 1, 2 or 3). If 2 people observed a sp. I divided counts/2, if 3 people observed it, I divided counts/3
There are different sample sizes because I calculated insects that were present. I did not include zeros (absent) when calculating averages.

Laurel Treviño Murphy 2-9-2021
