# Supplementary material for: The Native Bees of Texas: Evaluating the Benefits of a Public Engagement Course
Source: Insects. 2021 Aug 5;12(8):702. doi: 10.3390/insects12080702 (PMC8396608; doi:10.3390/insects12080702)
Supplement: Supplementary file 1 [file insects-12-00702-s001.zip › Native Bees of Texas Data Available Public Archive.zip!/Native Bees of Texas Course_Insect Survey_Blue Team_2019.pdf]

Fixed Route Survey 90 x 2 m curved path ID/counted flower visitors on flowers steady pace one direction 15 min

BLUE TEAM 2-year expert, instructor (students: S & J)
Start 2:15 End 2:30 p.m.

INSECT	BEE GROUP	COUNT (tally e.g. ~~IIII~~ III)	SUM (e.g. 5)	TOTAL INSECT COUNTS
BEE<br>Little or no hair on underside of abdomen<br><br>(pollen on leg hair or elsewhere)	Honey bee (Apis mellifera)	~~IIII~~ ~~IIII~~ I	11	
	Bumble bee (Bombus)	IIII	4	
	Large carpenter bee (Xylocopa virginica)	~~IIII~~ II	7	Xylocopa 8
	Large carpenter bee (Xylocopa micans)	I	1	
	Hairy leg/Longhorn (Diadasia/Melissodes)	III	3	
	Green sweat bee (Agapostemon)		0	
	Striped sweat bee (Halictus)		0	
	Tiny dark bee (Lasioglossum Hal./Ceratina Apid.)		0	
	Striped abdomen - miner		0	
Brush of hair under abdomen carries pollen	Striped abdomen - plasterer		0	
	Striped abdomen - oil-collector		0	
	Hairy belly bee / Striped leafcutter (Megachile)	~~IIII~~ III	8	
	Metallic hairy belly bee – mason (Osmia)		0	
No hair	Cuckoo/parasitic bee		0	
	Unidentified native bee		0	
	6 Bee species, 5 Native bee species	BEE COUNT	34	34 Bees – 11 HB = 23 NB
NON-BEE	Flies (Bombyliid)	IIII	4	
	Wasps (Mexican honey)	I	1	
	Butterflies/Moths (Skipper)	~~IIII~~ I	6	
	3 species non-bee insects	NON-BEE COUNT	11	11 non-bees of 45 insects
	9 species flower-visiting insects	INSECT COUNT	45	34 bees of 45 insects

Observer-Recorder Names: S & J (Blue team with 2-year expert)
Start 2:15 End 2:30 p.m.

INSECT	BEE GROUP	COUNT (tally e.g. 卌 III)	SUM (e.g. 5)	TOTAL INSECT COUNTS
BEE<br>Little or no hair on underside of abdomen<br><br>(pollen on leg hair or elsewhere)	Honey bee (Apis mellifera)		0	
	Bumble bee	卌 卌 卌 II	17	
	Large carpenter bee		0	
	Hairy leg bee / Longhorn		0	
	Green sweat bee	II	2	
	Striped sweat bee		0	
	Tiny dark bee		0	
	Striped abdomen - miner		0	
Brush of hair under abdomen carries pollen	Striped abdomen - plasterer		0	
	Striped abdomen - oil-collector		0	
	Hairy belly bee / striped leafcutter		0	
	Metallic hairy belly bee		0	
No hair	Cuckoo bee / parasitic bee			
	Unidentified native bee	卌 卌 卌 卌 卌 卌 卌 卌 卌<br>卌 卌 卌 卌 卌 卌 卌 卌 III	88	Dropped
	3 Bee species, 3 Native bee species	BEE COUNT	107	0 HB, 107 NB
	Flies	I	1	
NON-BEE	Wasps	卌	5	
	Butterflies/Moths		0	
	UFI		0	
	2 species non-bee insects	NON-BEE COUNT	6	6
	5 species flower-visiting insects	INSECT COUNT	113	

*Students said: “they weren’t honey bees, but we didn’t know what they were” (category Unidentified native bees)
