# Supplementary material for: The Native Bees of Texas: Evaluating the Benefits of a Public Engagement Course
Source: Insects. 2021 Aug 5;12(8):702. doi: 10.3390/insects12080702 (PMC8396608; doi:10.3390/insects12080702)
Supplement: Supplementary file 1 [file insects-12-00702-s001.zip › Native Bees of Texas Data Available Public Archive.zip!/Native Bees of Texas Course_Insect Survey_Red Team_2019.pdf]

Fixed Route Survey 90 x 2 m curved path ID/counted flower visitors on flowers steady pace one direction 15 min

RED TEAM 20-year expert (with S & C)
Start: 2:00 End: 2:15 p.m.

INSECT	BEE GENUS / SPECIES	COUNT (tally e.g. 𝍸 III)	SUM (e.g. 5)	TOTAL INSECT COUNTS
	Apis mellifera (honey bee)	𝍸 I	6	
	Bombus pensylvanicus (bumble bee)	𝍸 I	6	
	Xylocopa virginica (carpenter bee)	𝍸	5	(2 carpenter spp)
	Xylocopa micans (carpenter bee)	I	1	
	Diadasia enavata (hairy leg/longhorn)	I	1	
BEE	Melissodes (hairy leg/longhorn)	𝍸 II	7	
	Megachile exile (hairy belly/leafcutter)	𝍸 II	7	(2 leafcutter spp)
	Megachile sp. (hairy belly/leafcutter)	𝍸	5	
	Anthidiellum	III	3	
	Centris atripes	I	1	
	Coelioxys sp (cuckoo bee)	I	1	
	11 Bee species, 10 Native bee species	BEE COUNT	43	43 Bees - 6 HB = 37 NB
	Brachygastra mellifica (Honey wasp)	I	1	
NON-BEE	Skipper butterfly	𝍸 I	6	
	Butterfly	II	2	
	3 species non-bee insect	NON-BEE COUNT	9	9 non-bees of 52 insects
14 species all flower-visiting insects		INSECT COUNT	52	43 bees of 52 insects

NATIVE BEE COURSE FLOWER-VISITING INSECT SURVEY DATA SHEET 2019

Observer-Recorder Names: S & C (Red Team with 20-year expert)
Start: 2:00 End: 2:15 p.m.

INSECT	BEE GROUP	COUNT (tally e.g. ~~IIII~~ III)	SUM (e.g. 5)	TOTAL INSECT COUNTS
BEE<br>Little or no hair on underside of abdomen<br><br>(pollen on leg hair or elsewhere)	Honey bee (Apis mellifera)	~~IIII~~ ~~IIII~~ ~~IIII~~	15	
	Bumble bee	~~IIII~~ ~~IIII~~ ~~IIII~~ II	17	
	Large carpenter bee	I	1	
	Hairy leg bee / Longhorn	I	1	
	Green sweat bee		0	
	Striped sweat bee		0	
	Tiny dark bee		0	
	Striped abdomen - miner		0	
Brush of hair under abdomen carries pollen	Striped abdomen - plasterer		0	
	Striped abdomen - oil-collector		0	
	Hairy belly bee / striped leafcutter		0	
	Metallic hairy belly bee		0	
No hair	Cuckoo bee / parasitic bee		0	
	Unidentified native bee	~~IIII~~ IIII	9	
	4 Bee species, 3 Native bee species	BEE COUNT		43 TOT – 15 HB = 28 NB
NON-BEE	Flies	~~IIII~~ I	6	
	Wasps	I	1	
	Butterflies/Moths	~~IIII~~ ~~IIII~~ I	11	
	UFI			
	3 species non-bee insects	NON-BEE COUNT		18
	7 species flower-visiting insects	INSECT COUNT		61

Fixed Route Survey 90 x 2 m curved path ID/count flower visitors on flowers steady pace one direction 15 min

Location (Town, City) Lat/Long	Wildflower Center, Austin, TX Lat. 30° 11' 7.60" N Long. -97° 52' 23.56" W
Date (dd/MMM/yyyy)	Saturday, 05/OCT/2019
Time (CST) (circle a.m. or p.m.)	Start: 2;00 End: 2:15 a.m. | p.m.
Ambient air (circle one)	Cool | Warm | Hot
Skies (circle one)	Clear | Partly cloudy | Cloudy-bright overcast
Wind: Beaufort scale 0-4 (circle one)	Smoke rises (0) | Leaves rustle (1) | You feel wind on face (2) | Leaves move (3) | Small branches move (4)

Simplified Vegetation Survey: check veg. class, list dominant plants, estimate amount of inflorescences

Site Vegetation	☑	Dominant Plants, common name (daisy, sage, legume…)	Few (1-50)	Moderate (50-200)	Many (200-500)
Open Woodland					
Shrub-land					
Grassland / Prairie					
Gardens (native/not)	☑	Native Texas Hill Country wildflowers, grasses<br>Salvia farinacea, Ageratina, Pavonia, Wedellia,			☑
Agricultural crops					

Bio-Labs, University of Texas, Austin, TX Lat. 30°17'10.10" N, Long. -97°44'21.91" W
