# Supplementary material for: The Native Bees of Texas: Evaluating the Benefits of a Public Engagement Course
Source: Insects. 2021 Aug 5;12(8):702. doi: 10.3390/insects12080702 (PMC8396608; doi:10.3390/insects12080702)
Supplement: Supplementary file 1 [file insects-12-00702-s001.zip › Native Bees of Texas Data Available Public Archive.zip!/Native Bees of Texas Course_Insect Survey_Yellow Team_2019.pdf]

YELLOW TEAM: 3-year-expert (with J & S, students)
Start: 2:30 End: 2:45 p.m.

INSECT	BEE GROUP	COUNT (tally e.g. ~~IIII~~ III)	SUM (e.g. 5)	TOTAL INSECT COUNTS
BEE<br>Little or no hair on underside of abdomen<br><br>(pollen on leg hair or elsewhere)	Honey bee (Apis mellifera)	~~IIII~~ ~~IIII~~ ~~IIII~~ ~~IIII~~ ~~IIII~~ ~~IIII~~	30	
	Bumble bee (Bombus)	~~IIII~~ ~~IIII~~ II	12	
	Large carpenter bee (Xylocopa virginica)	~~IIII~~ ~~IIII~~ I	11	
	Hairy leg/Longhorn (Centris)	~~IIII~~ III	8	11 Hairyleg/longhorn
	Hairy leg/Longhorn	III	3	
	Green sweat bee (Agapostemon)		0	
	Striped sweat bee (Halictus)		0	
	Tiny dark bee (Lasioglossum Hal./Ceratina Apid.)	I	1	
	Striped abdomen - miner		0	
Brush of hair under abdomen carries pollen	Striped abdomen - plasterer		0	
	Striped abdomen - oil-collector		0	
	Hairy belly bee / striped leafcutter	~~IIII~~ ~~IIII~~ ~~IIII~~ IIII	19	
	Metallic hairy belly bee – mason (Osmia)		0	
No hair	Cuckoo/parasitic bee		0	
	Unidentified native bee		0	
	7 Bee species, 6 Native bee species	BEE COUNT	84	84 Bees – 30 HB = 54 NB
NON-BEE	Fly (Bombyliidae)	III	3	
	Wasp	IIII	4	
	UFI		0	
	2 species non-bee insects	NON-BEE COUNT	7	7 non-bees of 91 insects
9 species flower-visiting insects		INSECT COUNT	91	84 bees of 91 insects

NATIVE BEES OF TEXAS COURSE FLOWER-VISITING INSECT SURVEY DATA SHEET 2019

YELLOW TEAM: Observer-Recorder Names: J & S (with 3-year expert)
Start: 2:30 End: 2:45 p.m.

INSECT	BEE GROUP	COUNT (tally e.g. ~~IIII~~ III)	SUM (e.g. 5)	TOTAL INSECT COUNTS
BEE<br>Little or no hair on underside of abdomen<br><br>(pollen on leg hair or elsewhere)	Honey bee (Apis mellifera)	~~IIII~~ ~~IIII~~ I	11	
	Bumble bee	~~IIII~~ III	8	
	Large carpenter bee	~~IIII~~	5	
	Hairy leg bee / Longhorn	II	2	
	Green sweat bee		0	
	Striped sweat bee		0	
	Tiny dark bee	I	1	
	Striped abdomen - miner		0	
Brush of hair under abdomen carries pollen	Striped abdomen - plasterer	III	3	
	Striped abdomen - oil-collector		0	
	Hairy belly bee / striped leafcutter	~~IIII~~ ~~IIII~~ ~~IIII~~	15	
	Metallic hairy belly bee		0	
No hair	Cuckoo bee / parasitic bee		0	
	Unidentified native bee		0	
	7 Bee species, 6 Native bee species	BEE COUNT	45	45 Bees – 11 HB = 34 NB
NON-BEE	Flies		0	
	Wasps	I	1	
	Butterflies/Moths		0	
	UFI			
	1 species non-bee insects	NON-BEE COUNT	1	1 non-bees of 46 insects
8 species flower-visiting insects		INSECT COUNT	46	45 bees of 46 insects
