# Supplementary material for: The Native Bees of Texas: Evaluating the Benefits of a Public Engagement Course
Source: Insects. 2021 Aug 5;12(8):702. doi: 10.3390/insects12080702 (PMC8396608; doi:10.3390/insects12080702)
Supplement: Supplementary file 1 [file insects-12-00702-s001.zip › Native Bees of Texas Data Available Public Archive.zip!/Native Bees of Texas Course_Test & Feedback Std Tables_2020.pdf]

Native Bees of Texas Course – Results – Data Processing and Analysis

2019 (corrected & checked again 5-20-21, 5-21-21)
Laurel Treviño processed and modified tables & graphs from Shelly Engelman TIDES Interns' reports
Laurel & Shelly reviewed problems that Laurel detected & corrected

PROBLEM: FEEDBACK FORM - Some questions were worded differently without changing their meaning. But wording differences are substantial in questions 1, 5, 6.

	2018 Form		2019
~~1~~	Explain the importance of native bees in ecosystems and agroecosystems	~~1~~	Understand the importance of native bees in ecosystems and agroecosystems
2	Distinguish native bees from flower visiting insects that look like them	2	Distinguish native bees from other flower visiting insects that look like them
3	Use a basic guide to ID common native bees in Texas gardens, parks, farms, wildlands	3	Identify common native bees of Central Texas with the help of the basic guides we used in this class
4	Use my foundation of bee classification to learn more about native bee diversity	4	Use this foundation of bee family taxonomy to learn more about the huge diversity of native bees on my own
~~5~~	Describe management practices to conserve native bee habitat given my knowledge of their nesting habits and diet	~~5~~	Use best management practices to conserve native bee habitat given my knowledge of bee nesting habits, life cycles, and plant preferences
~~6~~	Identify native prairie plants for native bees in Central Texas	~~6~~	Choose the best combination of native prairie plants for native bee gardens in Central TX

SOLUTION: Laurel re-worded & summarized Qs to make 2018 & 2019 match

Learning Objective Statements	2018 (n = 12)	2019 (n = 19)
As a result of this course, I'm better able to …	Mean	Mean
Distinguish native bees from flower visitors that look like them	4.50	4.26
ID common native bees of Central Texas with course guides	4.33	4.00
Use bee taxonomy to learn more about bee diversity	4.33	4.37
Importance of native bees in agro/ecosystems	4.75	4.58
Best management practices to conserve native bee habitat	4.50	4.74
Native plants used by native bees in Central Texas	4.25	4.53

Learning Objective Ratings	2018 (n = 12)							2019 (n = 19)						
	Mean	SE	(1)	(2)	(3)	(4)	(5)	Mean	SE	(1)	(2)	(3)	(4)	(5)
Distinguish insects	4.50	0.15	0%	0%	0%	50%	50%	4.26	0.13	0%	0%	5%	63%	32%
Identify native bees	4.33	0.14	0%	0%	0%	67%	33%	4.00	0.15	0%	0%	21%	58%	21%
Diversity Taxonomy	4.33	0.14	0%	0%	0%	67%	33%	4.37	0.16	0%	0%	11%	42%	47%
Ecosystem services	4.75	0.13	0%	0%	0%	25%	75%	4.58	0.12	0%	0%	0%	42%	58%
Conservation	4.50	0.15	0%	0%	0%	50%	50%	4.74	0.10	0%	0%	0%	26%	74%
Native bee plants	4.25	0.18	0%	0%	8%	58%	33%	4.53	0.14	0%	0%	5%	37%	58%
Overall Mean (n=6)	4.44	0.07	-	-	-	-	-	4.41	0.11	-	-	-	-	-
SE (SD/SQRT(n)) = 2.45	SD = 0.18			0.18/2.45 = 0.07				SD = 0.26			0.26/2.45 = 0.11			

PROBLEM: TEST question order was different in 2018, 2019 (2-5-2020)

I compared original 2018 quizzes with 2018 report & compared with the 2019 report. Below is the ORIGINAL question order used in 2018 & 2019 reports

Q.	Current order in combined results table (Shelly)<br>(Shelly's pooled table only has Q1, Q2, etc., no wording)	Quiz Key	Report 2019	Quiz 2018	Report 2018	Report Pre %	Report Post %
1	Fly & bee. Which is the fly?	1	1	1	1	90	97
2	Wasp & bee. Which is the wasp?	2	2	2	2	80	70
3	Bee & flies. Which is the bee?	3	3	3	3	53	77
4	Four Bees. Which is the honey bee?	4	4	4	4	47	73
5	What sex is this leafcutter bee?	5	5	5	5	43	87
6	Where do most native bees nest	6	6	8	8	53	93
7	What features can help distinguish a bee from a fly or wasp?	7	7	9	9	33	87
8	Why are bees important for ecosystem functions?	8	8	6	6	70	100
9	What pollination services do bees provide us?	9	9	7	7	73	97
10	Why are native bee populations declining?	10	10	10	10	80	93

SOLUTION: Laurel checked 2018 & 2019 quiz results & report tables several times to make this table. (5-20-21 checked again)

Q.	Accuracy of insect identification and bee knowledge<br>(original order in 2018)	2018 Pre-test	2018 Post-test	n = 11 p-value	2019 Pre-test	2019 Post-test	n = 19 p-value
1	Fly & bee. Which is the fly?	73%	91%	0.303	100%	100%	1.000
2	Wasp & bee. Which is the wasp?	73%	73%	1.000	84%	68%	0.187
3	Bee & flies. Which is the bee?	36%	73%	0.102	63%	79%	0.331
4	Bees. Which is the honey bee?	55%	82%	0.193	42%	68%	0.056
5	What sex is this leafcutter bee?	45%	91%	0.028*	42%	84%	0.007**
6 (8)	Where do most native bees nest?	64%	100%	0.035*	21%	95%	0.000**
7 (9)	What features help distinguish a bee from a fly or wasp?	55%	91%	0.068	53%	89%	0.005**
8 (6)	Why are bees important for ecosystem functions?	55%	73%	0.408	74%	100%	0.021*
9 (7)	What pollination services do bees provide us?	55%	100%	0.015*	84%	100%	0.083
10	Why are native bee populations declining?	91%	100%	0.363	74%	89%	0.083
	Overall average	60%	87%	0.009**	64%	87%	0.000**

Native Bees of Texas Course – Results – Data Processing and Analysis

Tables with new, standardized FINAL question order, all stats checked & correct (Laurel Trevino 4-16-20)

Laurel made table below with same question order in both years to make dumbbell/dot charts comparable side-by-side (May 2020)

	% Correct Responses Pre & Post-Course	2018, (n = 11)					2019, (n=19)				
	Test Questions	Correct Pre	Std. Error	Correct Post	Std. Error	p-value	Correct Pre	Std. Error	Correct Post	Std. Error	p-value
1	Fly & bee. Which is the fly?	73%	0.14	91%	0.09	0.303	100%	0.00	100%	0.00	1.000
2	Wasp & bee. Which is the wasp?	73%	0.14	73%	0.14	1.000	84%	0.09	68%	0.11	0.187
3	Bee & flies. Which is the bee?	36%	0.15	73%	0.14	0.102	63%	0.11	79%	0.10	0.331
4	Bees. Which is the honey bee?	55%	0.16	82%	0.12	0.193	42%	0.12	68%	0.11	0.056
5	What sex is this leafcutter bee?	45%	0.16	91%	0.09	0.028*	42%	0.12	84%	0.09	0.007**
6	What features help distinguish bees, flies, wasps?	55%	0.16	91%	0.09	0.068	53%	0.12	89%	0.07	0.005**
7	Where do most native bees nest?	64%	0.15	100%	0.00	0.035*	21%	0.10	95%	0.05	0.000**
8	Why are bees important for ecosystem functions?	55%	0.16	73%	0.14	0.408	74%	0.10	100%	0.00	0.021*
9	What pollination services do bees provide us?	55%	0.16	100%	0.00	0.015*	84%	0.09	100%	0.00	0.083
10	Why are native bee populations declining?	91%	0.09	100%	0.00	0.363	74%	0.10	89%	0.07	0.083
	Overall Mean	60%	0.05	87%	0.03	0.009**	64%	0.04	87%	0.02	0.000**

corrected (5-20-21 checked), (5-21-2021 checked)
