# Supplementary material for: The Native Bees of Texas: Evaluating the Benefits of a Public Engagement Course
Source: Insects. 2021 Aug 5;12(8):702. doi: 10.3390/insects12080702 (PMC8396608; doi:10.3390/insects12080702)
Supplement: Supplementary file 1 [file insects-12-00702-s001.zip › Native Bees of Texas Data Available Public Archive.zip!/Native Bees of Texas Course_Test & Feedback_2018.pdf]

SUMMARY TABLE - TEST RESULTS

| | | | | | | | | | | | | | | |
|---|---|---|---|---|---|---|---|---|---|---|---|---|---|---|
| data | | | 40 | 42 | 53 | | 43 | 44 | 45 | 46 | 47 | 48 | | |
| | | | n | % Correct | Std. error | p-value | a | b | c | d | e | Blank | | |
| c | Q1. Fly and bee. Which is the fly? | Pre | 11 | 73% | 0.14 | 0.303 | 73% | 27% | | | | 0% | 100% | 0% |
| q | | Post | 11 | 91% | 0.09 | | 91% | 0% | | | | 9% | 100% | 0% |
| d | Q2. Wasp and bee. Which is the wasp? | Pre | 11 | 73% | 0.14 | 1.000 | 27% | 73% | | | | 0% | 100% | 0% |
| r | | Post | 11 | 73% | 0.14 | | 27% | 73% | | | | 0% | 100% | 0% |
| e | Q3. Bee and flies. Which is the bee? | Pre | 11 | 36% | 0.15 | 0.102 | 18% | 45% | 36% | 0% | | 0% | 100% | 0% |
| s | | Post | 11 | 73% | 0.14 | | 18% | 0% | 73% | 0% | | 9% | 100% | 0% |
| f | Q4. Bees. Which are honey bees? | Pre | 11 | 55% | 0.16 | 0.193 | 55% | 0% | 36% | 9% | | 0% | 100% | |
| t | | Post | 11 | 82% | 0.12 | | 82% | 0% | 0% | 18% | | 0% | 100% | |
| g | Q5. What sex is this leafcutter bee? | Pre | 11 | 45% | 0.16 | 0.028 | 55% | 45% | | | | 0% | 100% | |
| u | | Post | 11 | 91% | 0.09 | | 9% | 91% | | | | 0% | 100% | |
| h | Q6. Why are bees important for ecosystem functions? | Pre | 11 | 55% | 0.16 | 0.408 | 45% | 0% | 0% | 55% | 0% | 0% | 100% | |
| v | | Post | 11 | 73% | 0.14 | | 27% | 0% | 0% | 73% | 0% | 0% | 100% | |
| i | Q7. What pollination services do bees provide us? | Pre | 11 | 55% | 0.16 | 0.015 | 36% | 0% | 0% | 55% | 9% | 0% | 100% | |
| w | | Post | 11 | 100% | 0.00 | | 0% | 0% | 0% | 100% | 0% | 0% | 100% | |
| j | Q8. Where do most native bees' nest? | Pre | 11 | 64% | 0.15 | 0.035 | 64% | 18% | 9% | 9% | 0% | 0% | 100% | |
| x | | Post | 11 | 100% | 0.00 | | 100% | 0% | 0% | 0% | 0% | 0% | 100% | |
| k | Q9. What features can help me distinguish a bee from a fly or wasp in my garden? | Pre | 11 | 55% | 0.16 | 0.068 | 9% | 18% | 0% | 55% | 18% | 0% | 100% | |
| y | | Post | 11 | 91% | 0.09 | | 0% | 0% | 0% | 91% | 9% | 0% | 100% | |
| l | Q10. Why are native bee populations declining? | Pre | 11 | 91% | 0.09 | 0.363 | 91% | 0% | 0% | 9% | | 0% | 100% | |
| z | | Post | 11 | 100% | 0.00 | | 100% | 0% | 0% | 0% | | 0% | 100% | |
| m | Overall | Pre | 11 | 60% | 0.05 | 0.009 | | | | | | | | |
| aa | | Post | 11 | 87% | 0.03 | | | | | | | | | |

Shelly Engelman, 2018

SUMMARY TABLE - TEST RESULTS

| Pre | | | | | | | | | | | |
|---|---|---|---|---|---|---|---|---|---|---|---|
| n | 11 | 11 | 11 | 11 | 11 | 11 | 11 | 11 | 11 | 11 | 11 |
| Question | 1 | 2 | 3 | 4 | 5 | 6 | 7 | 8 | 9 | 10 | average |
| % correct | 73% | 73% | 36% | 55% | 45% | 55% | 55% | 64% | 55% | 91% | 60% |
| a | 0.73 | 0.27 | 0.18 | 0.55 | 0.55 | 0.45 | 0.36 | 0.64 | 0.09 | 0.91 | |
| b | 0.27 | 0.73 | 0.45 | 0.00 | 0.45 | 0.00 | 0.00 | 0.18 | 0.18 | 0.00 | |
| c | 0.00 | 0.00 | 0.36 | 0.36 | 0.00 | 0.00 | 0.00 | 0.09 | 0.00 | 0.00 | |
| d | 0.00 | 0.00 | 0.00 | 0.09 | 0.00 | 0.55 | 0.55 | 0.09 | 0.55 | 0.09 | |
| e | 0.00 | 0.00 | 0.00 | 0.00 | 0.00 | 0.00 | 0.09 | 0.00 | 0.18 | 0.00 | |
| blank | 0.00 | 0.00 | 0.00 | 0.00 | 0.00 | 0.00 | 0.00 | 0.00 | 0.00 | 0.00 | |
| sum | 1 | 1 | 1 | 1 | 1 | 1 | 1 | 1 | 1 | 1 | |
| std dev | 0.47 | 0.47 | 0.50 | 0.52 | 0.52 | 0.52 | 0.52 | 0.50 | 0.52 | 0.30 | 0.18 |
| std error | 0.14 | 0.14 | 0.15 | 0.16 | 0.16 | 0.16 | 0.16 | 0.15 | 0.16 | 0.09 | 0.05 |

| Post | | | | | | | | | | | |
|---|---|---|---|---|---|---|---|---|---|---|---|
| n | 11 | 11 | 11 | 11 | 11 | 11 | 11 | 11 | 11 | 11 | 11 |
| Question | 1 | 2 | 3 | 4 | 5 | 6 | 7 | 8 | 9 | 10 | average |
| % correct | 91% | 73% | 73% | 82% | 91% | 73% | 100% | 100% | 91% | 100% | 87% |
| a | 0.91 | 0.27 | 0.18 | 0.82 | 0.09 | 0.27 | 0.00 | 1.00 | 0.00 | 1.00 | |
| b | 0.00 | 0.73 | 0.00 | 0.00 | 0.91 | 0.00 | 0.00 | 0.00 | 0.00 | 0.00 | |
| c | 0.00 | 0.00 | 0.73 | 0.00 | 0.00 | 0.00 | 0.00 | 0.00 | 0.00 | 0.00 | |
| d | 0.00 | 0.00 | 0.00 | 0.18 | 0.00 | 0.73 | 1.00 | 0.00 | 0.91 | 0.00 | |
| e | 0.00 | 0.00 | 0.00 | 0.00 | 0.00 | 0.00 | 0.00 | 0.00 | 0.09 | 0.00 | |
| blank | 0.09 | 0.00 | 0.09 | 0.00 | 0.00 | 0.00 | 0.00 | 0.00 | 0.00 | 0.00 | |
| sum | 1 | 1 | 1 | 1 | 1 | 1 | 1 | 1 | 1 | 1 | |
| std dev | 0.30 | 0.47 | 0.47 | 0.40 | 0.30 | 0.47 | 0.00 | 0.00 | 0.30 | 0.00 | 0.11 |
| std error | 0.09 | 0.14 | 0.14 | 0.12 | 0.09 | 0.14 | 0.00 | 0.00 | 0.09 | 0.00 | 0.03 |

SUMMARY TABLE - FEEDBACK - COURSE RATINGS, LEARNING

| data | | 23 | 24 | 33 | 26 | 27 | 28 | 29 | 30 | 208 | | |
|---|---|---|---|---|---|---|---|---|---|---|---|---|
| | | n | Mean | SE | Strongly Disagree (1) | Disagree (2) | Neutral (3) | Agree (4) | Strongly Agree (5) | | % | % |
| b | Informative | 11 | 5.00 | 0.00 | 0 | 0 | 0 | 0 | 100 | | 100 | 100 |
| c | Useful | 11 | 5.00 | 0.00 | 0 | 0 | 0 | 0 | 100 | | 100 | 100 |
| d | Engaged | 11 | 5.00 | 0.00 | 0 | 0 | 0 | 0 | 100 | | 100 | 100 |
| e | Explain the importance of native bees in ecosystems and agroecosystems | 12 | 4.75 | 0.13 | 0 | 0 | 0 | 25 | 75 | | 100 | 100 |
| f | Distinguish native bees from flying visitors that look like them | 12 | 4.50 | 0.15 | 0 | 0 | 0 | 50 | 50 | | 100 | 100 |
| g | Use basic guide to ID the common native bees in Texas gardens, parks, or wildlands | 12 | 4.33 | 0.14 | 0 | 0 | 0 | 67 | 33 | | 100 | 100 |
| h | Use my foundation of bee classification to learn more about native bee diversity | 12 | 4.33 | 0.14 | 0 | 0 | 0 | 67 | 33 | | 100 | 100 |
| i | Describe management practices to conserve native bee habitats given my knowledge of their nesting habits & diet | 12 | 4.50 | 0.15 | 0 | 0 | 0 | 50 | 50 | | 100 | 100 |
| j | Identify native prairie plants for native bees in Central Texas | 12 | 4.25 | 0.18 | 0 | 0 | 8 | 58 | 33 | | 100 | 92 |

SUMMARY TABLE - COURSE RATINGS

| | | | | | | | | | |
|---|---|---|---|---|---|---|---|---|---|
| n | 11 | 11 | 11 | 12 | 12 | 12 | 12 | 12 | 12 |
| mean | 5.00 | 5.00 | 5.00 | 4.75 | 4.50 | 4.33 | 4.33 | 4.50 | 4.25 |
| 1 | 0% | 0% | 0% | 0% | 0% | 0% | 0% | 0% | 0% |
| 2 | 0% | 0% | 0% | 0% | 0% | 0% | 0% | 0% | 0% |
| 3 | 0% | 0% | 0% | 0% | 0% | 0% | 0% | 0% | 8% |
| 4 | 0% | 0% | 0% | 25% | 50% | 67% | 67% | 50% | 58% |
| 5 | 100% | 100% | 100% | 75% | 50% | 33% | 33% | 50% | 33% |
| std dev | 0.00 | 0.00 | 0.00 | 0.45 | 0.52 | 0.49 | 0.49 | 0.52 | 0.62 |
| std error | 0.00 | 0.00 | 0.00 | 0.13 | 0.15 | 0.14 | 0.14 | 0.15 | 0.18 |
| Total | 100% | 100% | 100% | 100% | 100% | 100% | 100% | 100% | 100% |

TEST AND FEEDBACK PER PARTICIPANT (BEE NAMES = De-Identified)

**Pre**

| Name | Pre/Post | 1 | 2 | 3 | 4 | 5 | 6 | 7 | 8 | 9 | 10 | Average Pre | Occupation | Occupation | Occupation | Occupation | 1 | 2 | 3 | 4 | 5 | 6 | 7 | 8 | 9 | 10 | Overall Pre |
|---|---|---|---|---|---|---|---|---|---|---|---|---|---|---|---|---|---|---|---|---|---|---|---|---|---|---|---|
| CORRECT | | A | B | C | A | B | D | D | A | D | A | | | | | | | | | | | | | | | | |
| Bumble Bee | Pre | A | B | B | C | B | A | D | A | B | D | | | | | | 1 | 1 | 0 | 0 | 1 | 0 | 1 | 1 | 0 | 0 | 50% |
| Striped Sweat Bee | Pre | A | A | C | A | B | D | E | D | E | A | | Gardener | Homemaker | Photographer | Curious person | 1 | 0 | 1 | 1 | 1 | 1 | 0 | 0 | 0 | 1 | 60% |
| Sunflower Bee | Pre | A | B | B | C | A | D | A | B | D | A | | Curious person | | | | 1 | 1 | 0 | 0 | 0 | 1 | 0 | 0 | 1 | 1 | 50% |

**Post**

| | | 1 | 2 | 3 | 4 | 5 | 6 | 7 | 8 | 9 | 10 | | | | | 1 | 2 | 3 | 4 | 5 | 6 | 7 | 8 | 9 | 10 | Overall Post |
|---|---|---|---|---|---|---|---|---|---|---|---|---|---|---|---|---|---|---|---|---|---|---|---|---|---|---|
| CORRECT | | A | B | C | A | B | D | D | A | D | A | | | | | | | | | | | | | | | |
| Bumble Bee | Post | A | B | C | A | B | D | D | A | D | A | | | | | 1 | 1 | 1 | 1 | 1 | 1 | 1 | 1 | 1 | 1 | 1 |
| Striped Sweat Bee | Post | A | A | C | A | B | A | D | A | D | A | Gardener | Homemaker | Photographer | Curious person | 1 | 0 | 1 | 1 | 1 | 0 | 1 | 1 | 1 | 1 | 0.8 |
| Sunflower Bee | Post | blank | B | A | A | B | D | D | A | E | A | Curious person | | | | 0 | 1 | 0 | 1 | 1 | 1 | 1 | 1 | 0 | 1 | 0.7 |

| | | | | | | | | | | | | | | | | | | | | | | | | | | | | | | | | | | | | | | | | | | | | | | | | | | |
|---|---|---|---|---|---|---|---|---|---|---|---|---|---|---|---|---|---|---|---|---|---|---|---|---|---|---|---|---|---|---|---|---|---|---|---|---|---|---|---|---|---|---|---|---|---|---|---|---|---|---|
| Longhorn Bee | Pre | A | B | C | D | B | A | A | C | A | A | Student | | | 1 | 1 | 1 | 0 | 1 | 0 | 0 | 0 | 0 | 1 | 50% | Longhorn Bee | Post | A | B | C | A | B | A | D | A | D | A | Student | | 1 | 1 | 1 | 1 | 1 | 0 | 1 | 1 | 1 | 1 | 0.9 |
| Cactus Bee | Pre | A | B | C | A | A | D | D | A | D | A | Curious person | Student | | 1 | 1 | 1 | 0 | 1 | 1 | 1 | 1 | 1 | 1 | 90% | Cactus Bee | Post | A | A | C | A | B | D | D | A | D | A | Curious person | Student | 1 | 0 | 1 | 1 | 1 | 1 | 1 | 1 | 1 | 1 | 0.9 |
| Cellophane Bee | Pre | B | B | C | A | A | D | D | A | D | A | Gardener | Curious person | | 0 | 1 | 1 | 1 | 0 | 1 | 1 | 1 | 1 | 1 | 80% | Cellophane Bee | Post | A | B | blank | A | A | D | D | A | D | A | Gardener | Curious person | 1 | 1 | 0 | 1 | 0 | 1 | 1 | 1 | 1 | 1 | 0.8 |
| Oil collecting bee | Pre | B | A | A | C | A | D | A | A | B | A | | | | 0 | 0 | 0 | 0 | 0 | 1 | 0 | 1 | 0 | 1 | 30% | Oil collecting bee | Post | A | B | C | D | B | D | D | A | D | A | Gardener | Curious person | 1 | 1 | 1 | 0 | 1 | 1 | 1 | 1 | 1 | 1 | 0.9 |
| Green sweat bee | Pre | A | B | A | C | A | A | A | A | D | A | Teacher/professor | | | 1 | 1 | 0 | 0 | 0 | 0 | 0 | 1 | 1 | 1 | 50% | Green sweat bee | Post | A | B | C | A | B | D | D | A | D | A | Teacher/professor | | 1 | 1 | 1 | 1 | 1 | 1 | 1 | 1 | 1 | 1 | 1 |
| Orchard bee | Pre | A | A | B | A | B | A | D | A | D | A | Gardener | Curious person | | 1 | 0 | 0 | 1 | 1 | 0 | 1 | 1 | 1 | 1 | 70% | Orchard bee | Post | A | B | C | A | B | A | D | A | D | A | Gardener | Curious person | 1 | 1 | 1 | 1 | 1 | 0 | 1 | 1 | 1 | 1 | 0.9 |
| Tiny Da | Pre | A | B | B | A | B | A | D | A | D | A | Natur | Photog | Curious person | 1 | 1 | 0 | 1 | 1 | 0 | 1 | 1 | 1 | 1 | 80% | Tiny Da | Po | A | B | C | A | B | D | D | A | D | A | | | 1 | 1 | 1 | 1 | 1 | 1 | 1 | 1 | 1 | 1 | 1 |

| | | | | | | | | | | | | | | | | | | | | | | | | |
|---|---|---|---|---|---|---|---|---|---|---|---|---|---|---|---|---|---|---|---|---|---|---|---|---|
| rk<br>Be<br>e<br>S<br>m<br>all<br>Ca<br>rp<br>en<br>ter<br>Be<br>e | P<br>r<br>e | B | B | B | A | A | D | D | B | E | A | al<br>is<br>t<br><br>G<br>ar<br>d<br>e<br>n<br>er | ra<br>ph<br>er<br><br>En<br>vir<br>on<br>m<br>en<br>tal<br>ed<br>u. | 0 | 1 | 0 | 1 | 0 | 1 | 1 | 0 | 0 | 1 | 5<br>0<br>% |
| rk<br>Be<br>e<br>S<br>m<br>all<br>Ca<br>rp<br>en<br>ter<br>Be<br>e | s<br>t<br><br>P<br>o<br>s<br>t | A | A | A | D | B | D | D | A | D | A | G<br>ar<br>d<br>e<br>n<br>er | Environmen<br>tal educator | 1 | 0 | 0 | 0 | 1 | 1 | 1 | 1 | 1 | 1 | 0<br>.<br>7 |

FEEDBACK RAW DATA (PARTICIPANT DE-ID BY #)

| | Please rate this event on how informative it was, how useful the knowledge and skills will be to you, & how engaged you were as a participant | | | As a result of today's workshop, I am better able to… | | | | | | | | |
|---|---|---|---|---|---|---|---|---|---|---|---|---|
| Participant | Informative | Useful | Engaged | Explain the importance of native bees in ecosystems and agroecosystems | Distinguish native bees from flying visitors that look like them | Use basic guide to ID the common native bees in Texas gardens, farms, or wildlands | Use my foundation of bee classification to learn more about native bee diversity | Describe management practices to conserve native bee habitats given my knowledge of their nesting habits & diet | Identify native prairie plants for native bees in Central Texas | Briefly describe how you intend to use skills/knowledge that you learned in today's session in your practice/home? | The best part(s) of attending this event was/were: | This event could be improved by: |
| 1 | 5 | 5 | 5 | 4 | 5 | 4 | 5 | 5 | 4 | | | |
| 2 | 5 | 5 | 5 | 4 | 4 | 4 | 4 | 4 | 3 | Add background to pollinators | | Graph seems either wrong or many more samples were taken and logged of the bees without bare ground. As I read the graph, it suggests bees go up as ground is covered. Opposite of reality? |
| 3 | 5 | 5 | 5 | 4 | 4 | 4 | 4 | 4 | 4 | UT course project | | |

| | | | | | | | | | | | | |
|---|---|---|---|---|---|---|---|---|---|---|---|---|
| 4 | 5 | 5 | 5 | 5 | 5 | 4 | 4 | 4 | 4 | Reduce use of insecticides; grow pollinator garden | Exploring the gardens | Longer garden time |
| 5 | 5 | 5 | 5 | 5 | 5 | 5 | 4 | 4 | 4 | I intend to teach others about native bee populations & behaviors, and to plant native plants! | Learning about how bees build their nests, especially the separated | Talking a little bit more about identifying plants! |
| 6 | 5 | 5 | 5 | 5 | 4 | 4 | 4 | 4 | 4 | Teach others and improve habitat | Handouts, style of presentation/photos on screen | More sharing of info between participants |
| 7 | 5 | 5 | 5 | 5 | 5 | 5 | 5 | 5 | 5 | I can plant more bee specific plants | Learning from an expert and going outside | [heart with arrow going through it] |
| 8 | 5 | 5 | 5 | 5 | 5 | 4 | 4 | 5 | 5 | I'm converting a small lawn (500 ft2) to native TX wildflower meadow | ID and conservation | I wish this workshop were longer or multi-segment! |
| 9 | 5 | 5 | 5 | 5 | 4 | 5 | 4 | 5 | 5 | Increase diversity of bees in my yard | Nice mix of lecture, lab and field time | |
| 10 | | | | 5 | 4 | 4 | 5 | 5 | 4 | Keep more leaves on ground and use as mulch. Also plant more native plants | Being at the Wildflower Center. Learning more about the native life | I thought the class was great |
| 11 | 5 | 5 | 5 | 5 | 4 | 4 | 4 | 4 | 5 | | I didn't know that many bees were ground nesters! Makes a huge difference in how I will garden in the future - real aha moment | Nothing that could have been done by the presenter…weather could have cooperated |
| 12 | 5 | 5 | 5 | 5 | 5 | 5 | 5 | 5 | 4 | For master gardener training sessions lecture to garden clubs | All parts were extremely useful | an increased time, another couple of hours |
