# Supplementary material for: The Native Bees of Texas: Evaluating the Benefits of a Public Engagement Course
Source: Insects. 2021 Aug 5;12(8):702. doi: 10.3390/insects12080702 (PMC8396608; doi:10.3390/insects12080702)
Supplement: Supplementary file 1 [file insects-12-00702-s001.zip › Native Bees of Texas Data Available Public Archive.zip!/Native Bees of Texas Course_Test & Feedback_2019.pdf]

SUMMARY TABLES

Test		n	% Correct	Std. Error	t-test	A	B	C	D	E
Q1. Fly and Bee. Which is the fly?	Pre	19	100%	0.00	1.000	100%	0%			
	Post	19	100%	0.00		100%	0%			
Q2. Wasp and Bee. Which is the wasp?	Pre	19	84%	0.09	0.187	16%	84%			
	Post	19	68%	0.11		32%	68%			
Q3. Bee and Flies. Which is the bee?	Pre	19	63%	0.11	0.331	0%	37%	63%	0%	
	Post	19	79%	0.10		16%	5%	79%	0%	
Q4. Bees. Which are the honey bees?	Pre	19	42%	0.12	0.056	42%	16%	16%	26%	
	Post	19	68%	0.11		68%	16%	0%	16%	
Q5. What sex is the leafcutter bee?	Pre	19	42%	0.12	0.007	58%	42%			
	Post	19	84%	0.09		16%	84%			
Q6. Where do most native bees' nest?	Pre	19	21%	0.10	0.000	21%	21%	11%	37%	11%
	Post	19	95%	0.05		5%	95%	0%	0%	0%
Q7. What features help me distinguish a bee from a fly or wasp in my garden?	Pre	19	53%	0.12	0.005	11%	26%	0%	53%	11%
	Post	19	89%	0.07		0%	11%	0%	89%	0%
Q8. Why are bees important for ecosystem functions? Because they…	Pre	19	74%	0.10	0.021	0%	21%	5%	74%	0%
	Post	19	100%	0.00		0%	0%	0%	100%	0%
Q9. What pollination services do bees provide us?	Pre	19	84%	0.09	0.083	0%	0%	16%	84%	0%
	Post	19	100%	0.00		0%	0%	0%	100%	0%
Q10. Why are native bee populations declining?	Pre	19	74%	0.10	0.083	0%	74%	0%	26%	0%
	Post	19	89%	0.07		0%	89%	0%	11%	0%
Overall average	Pre	19	64%	0.04	0.000					
	Post	19	87%	0.02						

Shelly Engelman, 2019

NATIVE BEES OF TEXAS COURSE – TEST AND FEEDBACK - 2019

Retrospective Self-rating - Before/Now		n	Mean	Std. Error	t-test	Poor (1)	Fair (2)	Good (3)	Very Good (4)	Excellent (5)
I would rate my knowledge of native bees as…	Before	20	1.65	0.21	0.000	60%	20%	15%	5%	0%
	Now	20	3.35	0.18		0%	10%	55%	25%	10%

Retrospective Self-rating	Poor (1)	Fair (2)	Good (3)	Very Good (4)	Excellent (5)	Buffer
Before	60%	20%	15%	5%	0%	80%
Now	0%	10%	55%	25%	10%	10%

	Participants' Feedback	n	Mean	Std. Error	Strongly Disagree (1)	Disagree (2)	Neutral (3)	Agree (4)	Strongly Agree (5)
	1. Informative	19	4.95	0.05	0%	0%	0%	5%	95%
	2. Useful	19	4.74	0.13	0%	0%	5%	16%	79%
	3. Engaging	19	4.26	0.17	0%	0%	16%	42%	42%
	Understand the importance of native bees in ecosystems and agroecosystems.	19	4.58	0.12	0%	0%	0%	42%	58%
	Distinguish native bees from other flower visiting insects that look like them.	19	4.26	0.13	0%	0%	5%	63%	32%
	Identify common native bees of Central Texas with the help of the basic guides we used in this class.	19	4.00	0.15	0%	0%	21%	58%	21%
	Use this foundation of bee family taxonomy to learn more about the huge diversity of native bees on my own.	19	4.37	0.16	0%	0%	11%	42%	47%
	Use best management practices to conserve native bee habitat given my knowledge of bee nesting habits, life cycles, and plant preferences.	19	4.74	0.10	0%	0%	0%	26%	74%
	Choose the best combinations of native prairie plan species to make a native bee garden in Central Texas.	19	4.53	0.14	0%	0%	5%	37%	58%

Shelly Engelman, 2019

Demographics		
Gender	n	%
Female	14	67%
Male	7	33%
Other	0	0%
Total	21	100%

Age	n	%
18-24	1	5%
25-44	5	24%
45-54	6	29%
55+	9	43%
Total	21	100%

Ethnicity	n	%
White	21	100%
Hispanic or Latinx	0	0%
Black or African American	0	0%
Asian/Pacific Islander	0	0%
Multiracial	0	0%
Other	0	0%
Total	21	100%

Highest Degree	n	%
High school degree	1	5%
Associate degree	0	0%
Bachelor's degree	10	48%
Master's degree	9	43%
Professional degree	0	0%
Doctorate	1	5%
Other	0	0%

Total	21	100%

Employment	n	%
Employed full time	13	62%
Employed part-time	1	5%
Student	1	5%
Retired	4	19%
Homemaker	0	0%
Self-employed	0	0%
Other	2	10%
Total	21	100%

TEST RAW DATA - 2019

		Pre Raw Data										Pre (1, Correct vs. 0, Incorrect)											Post Raw Data										Post (1, Correct vs. 0, Incorrect)											Before	Now
ID	Name	Pre1	Pre2	Pre3	Pre4	Pre5	Pre6	Pre7	Pre8	Pre9	Pre10	Pre1	Pre2	Pre3	Pre4	Pre5	Pre6	Pre7	Pre8	Pre9	Pre10	Average	Post1	Post2	Post3	Post4	Post5	Post6	Post7	Post8	Post9	Post10	Post1	Post2	Post3	Post4	Post5	Post6	Post7	Post8	Post9	Post10	Average	Before participating, I would rate my knowledge of native bees as (1, Poor, 2, Fair, 3, Good, 4, Very Good, 5, Excellent)	Now, I would rate my knowledge of native bees as (1, Poor, 2, Fair, 3, Good, 4, Very Good, 5, Excellent
1		A	B	B	A	B	D	B	D	D	B	1	1	0	1	1	0	0	1	1	1	70%	a	b	c	a	b	b	b	d	d	b	1	1	1	1	1	1	0	1	1	1	90%	1	3
2		a	b	c	b	b	b	a	d	d	d	1	1	1	0	1	1	0	1	1	0	70%	a	a	c	a	b	b	d	d	d	b	1	0	1	1	1	1	1	1	1	1	90%	3	4
3		a	b	c	c	a	c	b	d	d	b	1	1	1	0	0	0	0	1	1	1	60%	a	b	c	d	a	b	d	d	d	b	1	1	1	0	0	1	1	1	1	1	80%	1	4
4		a	a	b	d	b	c	d	c	c	b	1	0	0	0	1	0	1	0	0	1	40%	a	a	c	d	b	b	d	d	d	b	1	0	1	0	1	1	1	1	1	1	80%	1	3
5		a	b	b	b	a	a	b	b	c	b	1	1	0	0	0	0	0	0	0	1	30%	a	b	c	a	b	b	b	d	d	b	1	1	1	1	1	1	0	1	1	1	90%	2	3
6		a	b	c	a	a	d	d	d	d	b	1	1	1	1	0	0	1	1	1	1	80%	a	a	b	a	a	b	d	d	d	b	1	0	0	1	0	1	1	1	1	1	70%	1	3
7		a	a	b	c	a	d	b	d	d	b	1	0	0	0	0	0	0	1	1	1	40%	a	a	c	a	b	b	d	d	d	b	1	0	1	1	1	1	1	1	1	1	90%	1	2
8		a	b	c	b	a	d	e	b	c	b	1	1	1	0	0	0	0	0	0	1	40%	a	b	a	a	b	a	d	d	d	b	1	1	0	1	1	0	1	1	1	1	80%	1	3
9		A	B	C	A	B	B	B	D	D	B	1	1	1	1	1	1	0	1	1	1	90%	a	b	a	a	b	b	d	d	d	b	1	1	0	1	1	1	1	1	1	1	90%	3	3

10	a	b	c	a	a	a	e	d	d	b	1	1	1	1	0	0	0	1	1	1	70%	a	b	c	a	b	b	d	d	d	b	1	1	1	1	1	1	1	1	1	1	100%	3	5
11	a	b	c	c	b	b	d	d	d	d	1	1	1	0	1	1	1	1	1	0	80%	a	b	c	d	b	b	d	d	d	d	1	1	1	0	1	1	1	1	1	0	80%	2	3
12	a	b	b	d	b	d	a	b	d	b	1	1	0	0	1	0	0	0	1	1	50%	a	b	c	a	a	b	d	d	d	b	1	1	1	1	0	1	1	1	1	1	90%	1	4
13	a	b	c	a	a	d	d	d	d	d	1	1	1	1	0	0	1	1	1	0	70%	a	b	c	b	b	b	d	d	d	b	1	1	1	0	1	1	1	1	1	1	90%	1	3
14	a	b	c	d	a	e	d	d	d	b	1	1	1	0	0	0	1	1	1	1	70%	a	b	c	b	b	b	d	d	d	b	1	1	1	0	1	1	1	1	1	1	90%	1	3
15	a	b	c	a	b	a	d	d	d	b	1	1	1	1	1	0	1	1	1	1	90%	a	b	c	a	b	b	d	d	d	b	1	1	1	1	1	1	1	1	1	1	100%	2	4
16	a	a	c	a	a	a	d	d	d	b	1	0	1	1	0	0	1	1	1	1	70%	a	b	c	a	b	b	d	d	d	b	1	1	1	1	1	1	1	1	1	1	100%	1	3
17	a	b	b	d	a	d	d	d	d	d	1	1	0	0	0	0	1	1	1	0	50%	a	a	c	b	b	b	d	d	d	d	1	0	1	0	1	1	1	1	1	0	70%	1	3
18	a	b	b	d	a	e	d	d	d	d	1	1	0	0	0	0	1	1	1	0	50%	a	a	a	a	b	b	d	d	d	b	1	0	0	1	1	1	1	1	1	1	80%	1	2
19	a	b	c	a	b	b	d	b	d	b	1	1	1	1	1	1	1	0	1	1	90%	a	b	c	a	b	b	d	d	d	b	1	1	1	1	1	1	1	1	1	1	100%	4	4
20																																											2	5

TEST RAW DATA – 2019

n	19	19	19	19	19	19	19	19	19	19	19	19	19	19	19	19	19	19	19	19	19	19	20	20
% Correct	100%	84%	63%	42%	42%	21%	53%	74%	84%	74%	64%	100%	68%	79%	68%	84%	95%	89%	100%	100%	89%	87%	1.65	3.35
a	100%	16%	0%	42%	58%	21%	11%	0%	0%	0%		100%	32%	16%	68%	16%	5%	0%	0%	0%	0%		60%	0%
b	0%	84%	37%	16%	42%	21%	26%	21%	0%	74%		0%	68%	5%	16%	84%	95%	11%	0%	0%	89%		20%	10%
c	0%	0%	63%	16%	0%	11%	0%	5%	16%	0%		0%	0%	79%	0%	0%	0%	0%	0%	0%	0%		15%	55%
d	0%	0%	0%	26%	0%	37%	53%	74%	84%	26%		0%	0%	0%	16%	0%	0%	89%	100%	100%	11%		5%	25%
e	0%	0%	0%	0%	0%	11%	11%	0%	0%	0%		0%	0%	0%	0%	0%	0%	0%	0%	0%	0%		0%	10%
	100%	100%	100%	100%	100%	100%	100%	100%	100%	100%		100%	100%	100%	100%	100%	100%	100%	100%	100%	100%		100%	100%
t-test	#DIV/0!	0.187	0.331	0.056	0.007	0.000	0.005	0.021	0.083	0.083	0.000												0.000	
std dev	0	0.37	0.50	0.51	0.51	0.42	0.51	0.45	0.37	0.45	0.19	0.00	0.48	0.42	0.48	0.37	0.23	0.32	0.00	0.00	0.32	0.09	0.93	0.81
std. error	0	0.09	0.11	0.12	0.12	0.10	0.12	0.10	0.09	0.10	0.04	0.00	0.11	0.10	0.11	0.09	0.05	0.07	0.00	0.00	0.07	0.02	0.21	0.18

CORRECT	1	2	3	4	5	6	7	8	9	10
Pre	a	b	c	a	b	b	d	d	d	b
Post	a	b	c	a	b	b	d	d	d	b

Shelly Engelman, 2019

NATIVE BEES OF TEXAS COURSE – TEST AND FEEDBACK - 2019

FEEDBACK FORM – COURSE RATINGS – LEARNING – APPLICATION – BEST ASPECTS – IMPROVEMENTS -- RAW DATA - 2019

	To what extent was the class…			As a result of today's workshop, I am better able to…						Open-ended responses		
ID	1. Informative	2. Useful	3. Engaging	4. Understand the importance of native bees in ecosystems & argoecosystems	5. Distinguish native bees from other flower visiting insects that look like them	6. Identify common native bees of Central Texas with the help of the basic guides we used in this class	7. Use this foundation of bee family taxonomy to learn more about the huge diversity of native bees on my own	8. Use best management practices to conserve native bee habitat given my knowledge of bee nesting habits, life cycles, and plant preferences	9. Choose the best combinations of native prairie plan species to make a native bee garden in Central Texas	10. Describe how you intend to use skills/knowledge that you learned in today's session in your practice/home	11. The best part of this event was/were	12. This event could be improved by
1	5	4	4	5	3	4	4	5	5		Everything; it was great!	N/A
2	5	5	3	5	4	4	4	4	4	Home landscaping & continue efforts with Texas Master naturalists	Bee observations under the microscope	N/A
3	5	5	5	4	4	4	4	5	3	Building habitat at our home	The labs	
4	4	3	3	4	4	3	3	4	4		All the good info	not reading slides word for word
5	5	5	4	5	4	4	5	5	5	ID Photo project		
6	5	5	3	4	5	5	5	5	5	I've been 40 years range science student & reestablishment of native grasses	bee-actual bees	more field time
7	5	5	4	5	4	3	4	4	4	Share knowledge' maybe plant some flowers	Learning about Bees! I found I knew nothing before	
8	5	5	5	5	4	4	5	5	5	Lawn and garden care and development	knowing about all the types of sizes of bees!	
9	5	5	5	5	4	3	4	5	5	increase native prairie & bee friendly wildflowers on my ranch	So much! Learning how to distinguish bees from flies/wasps; learning how to provide habitat	labeling the bees in the scopes or telling us the answers later

Shelly Engelman, 2019

10	5	5	5	4	5	4	5	5	5	As a master gardener, I teach an intro to native been class as well as a pollinator/insect habitat class	Seeing actual bees under scopes and learning how to better teach this topic. Thought it was great	
11	5	4	4	5	4	4	3	4	5	By creating a pollinator garden	Both lectures and lab complemented the learning process)	
12	5	5	5	5	5	5	5	5	5	Lang management/pollinators; presentation background for garden clubs & master gardeners	all of it	Longer time involvement; fall day (better weather)
13	5	4	5	5	4	4	4	4	4		Bee labs	
14	5	5	4	4	5	4	5	5	4	Identify what I have and plan to improve habitat	bee examples and excellent instructor	warmer room
15	5	5	4	5	5	5	4	5	4	Plant bee-friendly plants	excellent instructor!	warmer room and set lunch break
16	5	5	5	5	5	5	5	5	4	I'll try to grow as much as I can from an apartment	Learning on bee behavior	Include lunch
17	5	5	5	4	4	4	4	5	5	Increase flowers for native meadows	to be able to understand native bees and realize they are mostly solitary. [I realize that you] don't need honey bees to improve ecosystem	I could use less details about metamorphosis
18	5	5	4	4	4	3	5	5	5	Documenting; increasing number & variety of bees at the ranch		
19	5	5	4	4	4	4	5	5	5	I am converting our front & back yard to pollinator gardens	handouts/sliders	longer, slower pace
20												
21												

SUMMARY FEEDBACK FORM 2019

n	19	19	19	19	19	19	19	19	19
mean	4.95	4.74	4.26	4.58	4.26	4.00	4.37	4.74	4.53
1	0%	0%	0%	0%	0%	0%	0%	0%	0%
2	0%	0%	0%	0%	0%	0%	0%	0%	0%
3	0%	5%	16%	0%	5%	21%	11%	0%	5%
4	5%	16%	42%	42%	63%	58%	42%	26%	37%
5	95%	79%	42%	58%	32%	21%	47%	74%	58%
	100 %	100 %	100 %	100%	100%	100%	100%	100 %	100 %
stddev	0.23	0.56	0.73	0.51	0.56	0.67	0.68	0.45	0.61
stderr or	0.05	0.13	0.17	0.12	0.13	0.15	0.16	0.10	0.14

INTENDED USE OF ACQUIRED KNOWLEDGE – DATA - 2019

f	10. Describe how you intend to use skills/knowledge that you learned in today's session in your practice/home:
Educate Other	As a master gardener, I teach an intro to native been class as well as a pollinator/insect habitat class
Educate Other	continue efforts with Texas Master naturalists
Educate Other	Share knowledge' maybe plant some flowers
Educate Other	presentation background for garden clubs and master gardeners
Improve Garden/Land	Building habitat at our home
Improve Garden/Land	By creating a pollinator garden
Improve Garden/Land	Documenting; increasing number nad variety of bees at the ranch
Improve Garden/Land	Home landscaping
Improve Garden/Land	I am converting our front and back yard to pollinator gardens
Improve Garden/Land	Identify what I have and plan to improve habitat
Improve Garden/Land	I'll try to grow as much as I can from an apartment
Improve Garden/Land	Increase flowers for native meadows
Improve Garden/Land	increase native prairie and bee friendly wildflowers on my ranch
Improve Garden/Land	I've been a ranger for 40 years and continue to work to reestablish native grasses
Improve Garden/Land	Lang management/pollinators
Improve Garden/Land	Lawn and garden care and development
Improve Garden/Land	Plant bee-friendly plants
Project	ID Photo project

NATIVE BEES OF TEXAS COURSE – TEST AND FEEDBACK - 2019

NEEDS IMPROVEMENT – DATA - 2019

Category	Response
Lunch	Include lunch
	Set lunch break
Duration and Pace	Longer time involvement
	longer, slower pace
	more field time
	N/A
	N/A
More active learning	not reading slides word for word
	I could use less details about metamorphosis
	labeling the bees in the scopes or telling us the answers later
Climate	warmer room
	warmer room and set lunch break
	Better weather

BEST ASPECTS OF COURSE – DATA - 2019

Category	Response
General-positive	Everything; it was great!
General-positive	all of it
Information	All the good info
Information	Learning about Bees! I found I knew nothing before.
Information	knowing about all the types of sizes of bees!
Information	so much! Learning how to distinguish bees from flies/wasps; learning how to provide habitat
Information	Learning on bee behavior
Information	to be able to understand native bees and realize they are mostly solitary. [I realize that you] don't need honey bees to improve ecosystem
Instruction	Both lectures and lab complemented the learning process)
Instruction	bee examples and excellent instructor
Instruction	excellent instructor!
Lab and Garden Walk	bee-actual bees
Microscope Lab	Bee observations under the microscope
Microscope Lab	The labs
Microscope Lab	Seeing actual bees under scopes and learning how to better teach this topic. Thought it was great

Microscope Lab	Bee labs
Resources	handouts/sliders

DEMOGRAPHICS DATA - 2019

ID	Name	Gender	Age	Ethnicity	Highest degree or level of school completed?	Employment status	Occupation	Zip code	Notes
1		f	45-54	w	ma	full time		78641	
2		f	55+	w	bac	retired		78723	
3		f	25-44	w	bac	Other		78615	
4		f	45-54	w	ma	full time		78676	
5		f	55+	w	ma	full time		76825	
6		m	55+	w	ma	retired		78633	
7		m	55+	w	bac	full time		78676	
8		m	25-44	w	bac	full time		78626	acquired information via web & personal communication at workshop
9		f	55+	w	bac	full time		78628	acquired via LinkedIn
10		m	55+	w	doctorate	retired		78130	
11		m	18-24	w	high	student		78705	enrolled at UT Austin FRI bugs research stream; wanted to learn more about insects without taking a formal class at UT
12		f	25-44	w	ma	full time		78749	
13		f	45-54	w	ma	full time		78633	
14		m	45-54	w	bac	retired		78628	
15		f	55+	w	ma	part time		78610	
16		f	25-44	w	bac	full time		78751	
17		m	25-44	w	ma	Other		78615	
18		f	45-54	w	bac	full time		78666	
19		f	45-54	w	bac	full time		78628	
20		f	55+	w	ma	full time		78660	
21		f	55+	w	bac	full time		78626	acquired information via web & personal communication at workshop

n	21	21	21	21	21	1	21
f	67%	0%	0%	0%	0%	#REF!	0%
m	33%	0%	0%	0%	0%		
18-24		5%	0%	0%	0%		
25-44		24%	0%	0%	0%		
45-54		29%	0%	0%	0%		
55+		43%	0%	0%	0%		
w		0%	100%	0%	0%		
high				5%	0%		
bac				48%	0%		
ma				43%	0%		
doctorate				5%	0%		
full time					62%		
part time					5%		
retired					19%		
student					5%		
Other					10%		
	100%	100%	100%	100%	100%	#REF!	0%

Row Labels	Count of Zip code
76825	1
78130	1
78610	1
78615	2
78626	2
78628	3
78633	2
78641	1
78660	1
78666	1
78676	2
78705	1
78723	1
78749	1
78751	1
Grand Total	21

MAP ZIP CODES

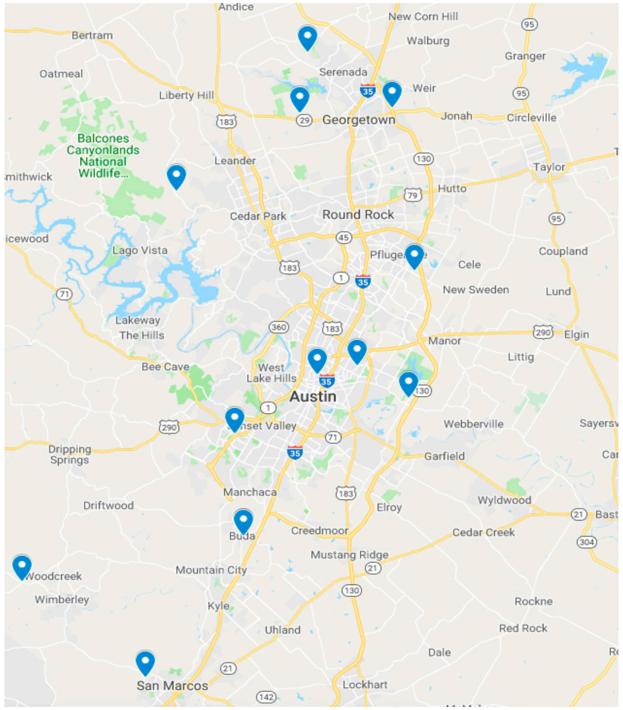

Shelly Engelman, 2019
